# Emerging roles of cancer-associated histone mutations in genomic instabilities

**DOI:** 10.3389/fcell.2024.1455572

**Published:** 2024-10-08

**Authors:** Priyanka Yadav, Ronit Jain, Rajesh Kumar Yadav

**Affiliations:** Cancer Epigenomics Laboratory, National Institute of Immunology, New Delhi, India

**Keywords:** chromatin, genomic instability, oncohistone, epigenetic mechanism, histone mutation, central nervous system, oncology

## Abstract

Epigenetic mechanisms often fuel the quick evolution of cancer cells from normal cells. Mutations or aberrant expressions in the enzymes of DNA methylation, histone post-translational modifications, and chromatin remodellers have been extensively investigated in cancer pathogenesis; however, cancer-associated histone mutants have gained momentum in recent decades. Next-generation sequencing of cancer cells has identified somatic recurrent mutations in all the histones (H3, H4, H2A, H2B, and H1) with different frequencies for various tumour types. Importantly, the well-characterised H3K27M, H3G34R/V, and H3K36M mutations are termed as oncohistone mutants because of their wide roles, from defects in cellular differentiation, transcriptional dysregulation, and perturbed epigenomic profiles to genomic instabilities. Mechanistically, these histone mutants impart their effects on histone modifications and/or on irregular distributions of chromatin complexes. Recent studies have identified the crucial roles of the H3K27M and H3G34R/V mutants in the DNA damage response pathway, but their impacts on chemotherapy and tumour progression remain elusive. In this review, we summarise the recent developments in their functions toward genomic instabilities and tumour progression. Finally, we discuss how such a mechanistic understanding can be harnessed toward the potential treatment of tumours harbouring the H3K27M, H3G34R/V, and H3K36M mutations.

## 1 Introduction

Cells of the same kind have the same genetic information and genes, but only a small subset of them are transcribed at any given time. Among the many regulatory pathways, it is possible that epigenetic mechanisms can cause heritable changes in gene expressions without altering the genetic sequence, thereby transforming transient signalling events into long-term changes in organism performance. The DNA encodes genetic information within nucleosomal arrays to form chromatin (Talbert and Henikoff, 2010; Allis and Jenuwein, 2016). In eukaryotic cells, the DNA is wrapped around a histone octamer containing two copies of each core histone, namely H2A, H2B, H3, and H4, to form nucleosomes. In the nucleosomes, unstructured N-terminal tails extend outward and are subjected to post-translational modifications (PTMs). Each amino acid and PTM on the small tails of the histones uniquely determine the nucleosome structure and impact the functions of the proteins that add the PTMs (writers), recognise the PTMs (readers), and remove the PTMs (erasers). Additionally, histone variants such as the H3.3 add further complexities to the eukaryotic epigenome by regulating the chromatin structure functions (Kallappagoudar et al., 2015; Maze et al., 2014; Cohen and Meshorer, 2024). Within the nucleus, the organisation of the chromatin into higher-order structures enables formation of chromatin domains that carry out the diverse cellular signalling functions, including gene expression regulation and providing a conducive chromatin environment. The dynamic PTMs of histones, such as acetylation, methylation, and phosphorylation, serve as key mediators of signalling events by controlling DNA accessibility for the assembly of writers, readers, erasers, and chromatin remodeller proteins. Furthermore, PTMs are tightly regulated by the complex interplay of transcription factors, chromatin-modifying complexes, and signalling pathways, which in turn integrate precise control of the gene expression upon developmental cues with environmental stimuli. To develop stable and reversible epigenomic phenotypes, epigenetic signals from the developmental and environmental cues must be centrally integrated at the nucleus (Flavahan et al., 2017). Perturbations of the chromatin structure have profound effects on cells through alteration of gene expressions or initiation of genomic instabilities (Jones and Baylin, 2007).

Chromatin is not only the packaging material of the DNA but also the orchestrator of signalling events whenever cells experience intrinsic or extrinsic DNA damage (Dabin et al., 2023; Yao and Dai, 2014; Jackson and Bartek, 2009). Therefore, in response to DNA lesions, multiple cellular pathways regulate the DNA damage response (DDR) network to sense, signal, and repair the DNA lesions in the context of chromatin (Dabin et al., 2023; Jackson and Bartek, 2009; Arnould et al., 2023; Pinto et al., 2021; Ferrand et al., 2020). Furthermore, chromatin-modifying complexes, chromatin remodellers, and histone PTMs bridge the DNA lesions with the DDR pathways by including DNA damage and mitotic checkpoint proteins. Their dysregulation can cause genome instabilities and mutations that drive cancer cell development, allowing cell clones and cell-to-cell variations both inside tumours and between the tumour and its metastasis (Shen and Laird, 2013). Inactivating mutations in the epigenomic components can disrupt gene expression and genomic stability through DNA methylation–demethylation reactions, PTMs, and alteration of the positioned nucleosomes (You and Jones, 2012).

In this review, we summarise the most recent data on how oncohistones contribute to tumour development mechanistically through their strong impacts on the chromatin states, gene expressions, and genomic stability. Recent studies have reported how various histone mutations impair the chromatin structure–function relationships, resulting in genomic instabilities, dysregulated DDR pathway activation, and defects in DNA repair (Giacomini et al., 2024; Rominiyi and Collis, 2022). However, more precise studies are required to establish the mechanisms and identify the mutation-specific functions of oncohistones in genome instability and the DDR pathways. Finally, we also discuss how combinatorial inhibitors in the chromatin modulator and DDR network could provide opportunities for treating cancers involving H3K27M, H3G34R/V, and H3K36M mutations. By compiling findings from various studies, this review aims to provide a comprehensive understanding that would enable new therapeutic approaches.

## 2 Methodology

Histone mutations in cancer were discovered in 2012 through genome sequencing events. We searched for cancer-associated histone mutations from 2012 to January 2024 on PubMed, Google search, and the cancer genome database using the following phrases: ‘histone mutation in cancer’, ‘histone H3 in cancer’, ‘H3K27M’, ‘H3G34R’, ‘H3G34V’, ‘H3G34W’, ‘H3K36M’, ‘oncohistone’, ‘histone mutation in glioma’, ‘histone mutation and brain tumour’, ‘histone mutation in bone tumour’, ‘oncohistone and glioblastoma’, ‘cancer-associated histone mutants in genomic instability or DNA damage’, ‘H3K27M or H3G34R/V’, and ‘genomic instability or DNA damage’. All resulting articles and reviews were critically analysed to summarise the data without using specific inclusion/exclusion criteria. However, the main focus of this review was to identify the emerging roles of cancer-associated histone mutations in genomic instabilities. To ensure simplicity and a mechanistic understanding, the specific focus of this review is on cancer-associated histone H3 mutations, while other histone mutants are excluded. For simplicity, we also include a mechanistic analysis of the H3.3G34R, H3.3G34V, and H3.3K27M mutants in tumorigenesis as a consequence of gene expression changes or the impacts of genomic instabilities. Readers are also referred to another review on histone localisation and nomenclatures regarding chromosomes (Amatori et al., 2021).

## 3 How do histone mutations contribute to cancer?

Following the discovery of the human genome sequence, numerous studies have found genomic aberrations in cancer cells through next-generation sequencing. Before 2012, aberrant gene expressions or mutations were identified in the writers, erasers, readers, and chromatin remodeller proteins of various epithelial, haematological, and other cancers (Shen and Laird, 2013), but mutations in the histones themselves are now emerging as a common feature of many cancers (Bonner et al., 2023; Sahu and Lu, 2022; Hanahan, 2022).

### 3.1 Histone H3 is mutated in various cancers

Among the solid tumours, those developing in the brain and central nervous system (CNS) (approximately 100 types) have caused the most cancer-related deaths in children (Capper et al., 2018; Downing et al., 2012; Louis et al., 2021). The major difference between adult and paediatric brain and solid tumours is the tissue development and organogenesis determining the molecular characteristics of tumours (Downing et al., 2012). Country-wise surveys indicate that tumours occurring in the CNS vary from 1.12 to 5.14 cases per 100,000 persons per country. Among these, paediatric high-grade gliomas (pHGGs) constitute approximately 10% of the tumours, and about 40% of the deaths occur due to pHGGs including glioblastomas and diffuse intrinsic pontine gliomas (DIPGs) (Louis et al., 2021; Johnson et al., 2014; Moudgil-Joshi et al., 2021; Louis et al., 2016). Standard care for adult HGGs include resection, radiotherapy, and temozolomide (TMZ) administration; however, this course of cancer management does not work properly with pHGGs as it is clear that the molecular basis of pHGG is different from that of adult HGG. Even though the histology of paediatric glioma is similar to that of adult glioma, the advent of next-generation sequencing, proteomics, and epigenetic profile analyses have led researchers to classify pHGGs into many subtypes (Louis et al., 2021). pHGGs are aggressive cancers with poor survival rates (Ocasio et al., 2023); pHGGs and their adult counterparts can be distinguished by molecular subtyping for histone mutational status in neuro-oncology (Louis et al., 2021; Moudgil-Joshi et al., 2021; Faury et al., 2007; Mackay et al., 2017). For example, diffuse midline glioma (DMG) with H3K27 mutation is a novel type of CNS tumour declared by the World Health Organization (Louis et al., 2016). Herein, we discuss the prevalence of histone mutations in various cancers.

#### 3.1.1 Puzzling piece of histone mutations: tissue-specific or multiple tissue prevalence?

**FIGURE 1:**
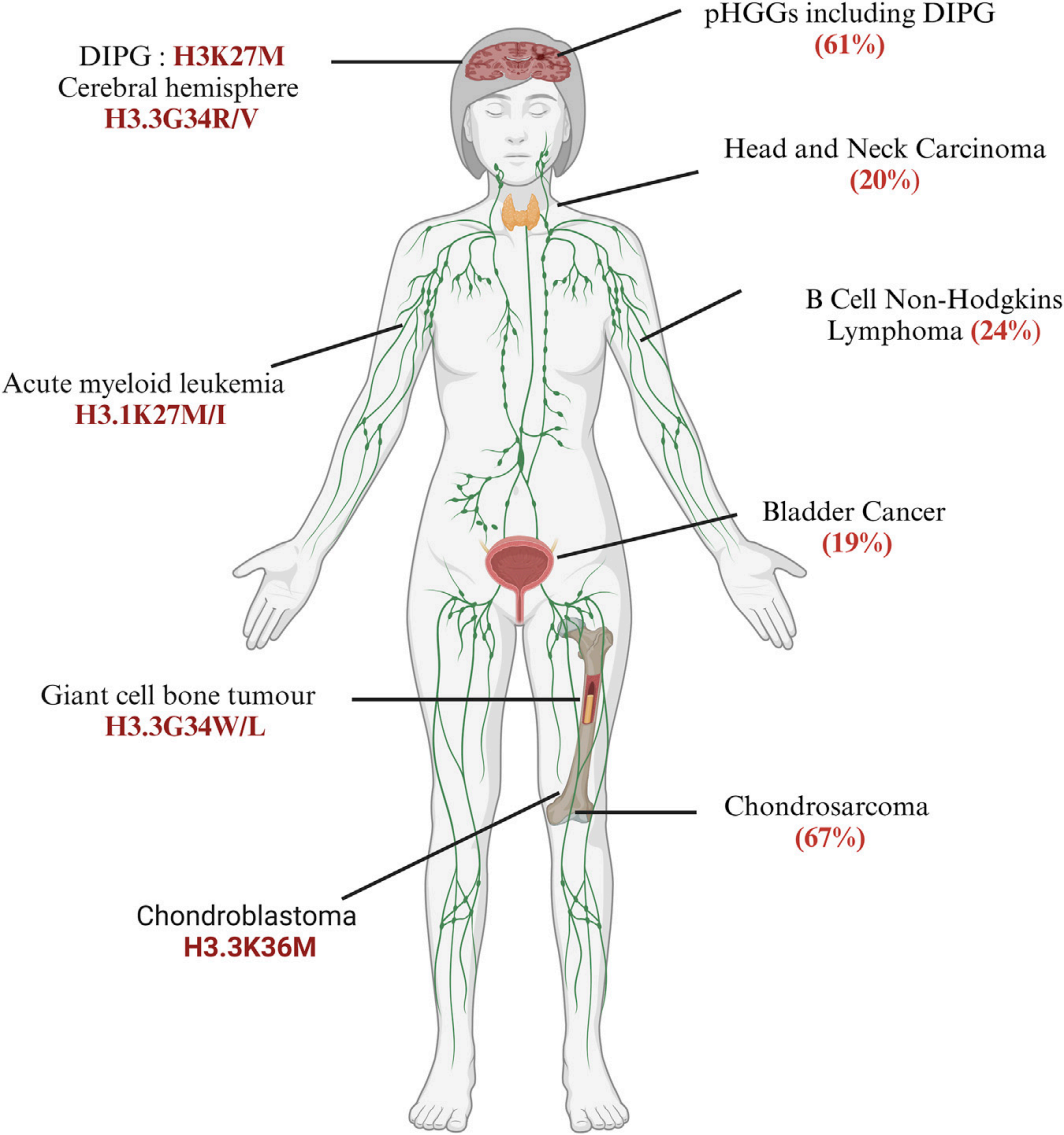
Frequency and distribution of somatic histone mutations across different cancer types: the left panel indicates specific histone H3 mutants discussed in this review, while the right panel mentions the total frequency of core histones in patients, as discussed by Bonner et al. (2023). Diffuse intrinsic pontine gliomas (DIPGs) (created with BioRender.com, accessed on 9 June 2024).

pHGGs consist of 80% of DMGs, which include both DIPGs and other diffuse gliomas harbouring missense mutations of lysine-27 to methionine (H3.3K27M); however, the cerebral hemispheres of older adolescents and young adults harbour approximately 30% of diffuse gliomas with glycine-34 to arginine or valine (H3.3G34R/V) mutations encoded mainly by the *H3F3A* gene (Ocasio et al., 2023; Khuong-Quang et al., 2012; Wu et al., 2012; Schwartzentruber et al., 2012; Taylor et al., 2014; Fontebasso et al., 2014). Notably, the H3.3G34R and H3.3K27M mutations are mutually exclusive, and their localisation and expressions of region-specific neurodevelopmental signatures are quite different in the brain (Mackay et al., 2017; Khuong-Quang et al., 2012; Bressan et al., 2021; Sturm et al., 2012). H3.3G34 tryptophan (W) and H3.3G34 leucine (L) are found in the giant cell tumours of the bone (GCTB) (Behjati et al., 2013) but not in CNS tumours; furthermore, 11% of paediatric primitive neuroectodermal tumours of the CNS (CNS-PNETs) have the H3.3G34R mutation (Gessi et al., 2013). Paediatric patients with glioblastomas and CNS-PNETs should be diagnosed carefully. Interestingly, in adults, H3.3K27M was found only in the *H3F3A* gene but not in other histone H3 genes (Schulte et al., 2020). The histone H3 variant H3.3 protein also encoded by *H3F3B* has a lysine-36 to methionine (H3.3K36M) mutation in 90% of chondroblastomas (Behjati et al., 2013; Fang et al., 2016) and glycine-34 to tryptophan/leucine (H3.3G34W/L) in GCTBs. These oncohistones broadly contribute to tumour development by impacting gene expressions and the regulatory attributes of cellular differentiation (Sahu and Lu, 2022; Mohammad and Helin, 2017). Further cataloguing of cancers has enabled the identification of an expanding landscape of ‘oncohistone’ mutations in various human cancers (11% of tumours having somatic histone mutations) (Bonner et al., 2023; Nacev et al., 2019). Overall, the estimation of all cancers including those in children, young adults, and adults show that the highest prevalence rates were found in 67% of chondrosarcomas, more than 60% of pHGGs, and 30% of lymphomas (Bonner et al., 2023) (Figure 1). Identification of H3K27M in medulloblastoma has serious implications for the diagnostic value of cancer subtyping (Dottermusch et al., 2022). In posterior fossa A (PFA) ependymomas, one subtype of ependymomas (EPN) is rarely mutated for H3.3K27M but these tumours have increased expressions of the enhancer of zeste homologue inhibitory protein (EZHIP), which causes reduced H3K27 methylation like in H3K27M, suggesting the importance of H3K27 residues as hotspot mutations in brain tumorigenesis (Jenseit et al., 2022; Hubner et al., 2019; Ryall et al., 2017). Outside the CNS, H3.1K27M or H3.1K27I mutations are frequently found in acute myeloid leukaemia (AML) but not in H3.3 proteins (Lehnertz et al., 2017). It is clearly noted that DMGs harbouring H3.1K27M and H3.3K27M contribute differently to tumorigenesis based on the epigenomic profile and transcriptional status (Zhang et al., 2024; Liu et al., 2022; Jessa et al., 2022; Castel et al., 2015). These studies suggest that histone H3 mutations are both tissue-specific and tissue-independent, meaning that the same mutants may be found in other tissues as well (Figure 1). This fact will be further discussed in the next section through the mechanistic aspects of histone mutations.

### 3.2 H3K27M, H3G34R/V, and H3K36M mutations alter the chromatin states in several cell types

To understand the impacts of histone mutations on the epigenome of cancer cells, several model systems have been utilised for *in vitro* studies to *in vivo* characterisations using tissue cultures, patient-derived samples, mouse models, and *Drosophila* to eukaryotic yeast systems, for which the readers are referred to other works (Zhang et al., 2023; Chaouch and Lasko, 2021). Herein, we summarise the data obtained in the context of tissue cultures, mouse models, and xenograft models to establish whether histone mutants contribute to tumorigenesis alone or in combination with other factors. We also correlate these findings with those from other eukaryotic model systems to understand the biology of the histone mutant phenotypes independent of the heterogeneity of cancer cells wherever required.

#### 3.2.1 H3K27M alters PTMs at the H3K27 residue

The context of the epigenome in the developmental pathways is essentially a dynamic manifestation of the signalling molecules, growth factors, cellular memory, and differentiation cues to establish a faithful cellular context in organogenesis. Histones are just one component of a complex chromatin environment. In the evolutionary context, multiple alleles of histones are present in the cells to provide redundancy for cell survival. Ideally, deletions or a few mutations in the histones should not have dramatic impacts on cell survival or tumorigenesis. Interestingly, histone alterations in cancers occur in just one allele, and they are somatic heterozygous mutations. Therefore, the foremost thing is to determine whether the histone mutant acts dominantly in the presence of a wild-type (WT) copy of the histone. It is crucial to determine the contributions of histone mutations in tumour development by understanding the altered landscape of the epigenome in cancer cells.

**FIGURE 2:**
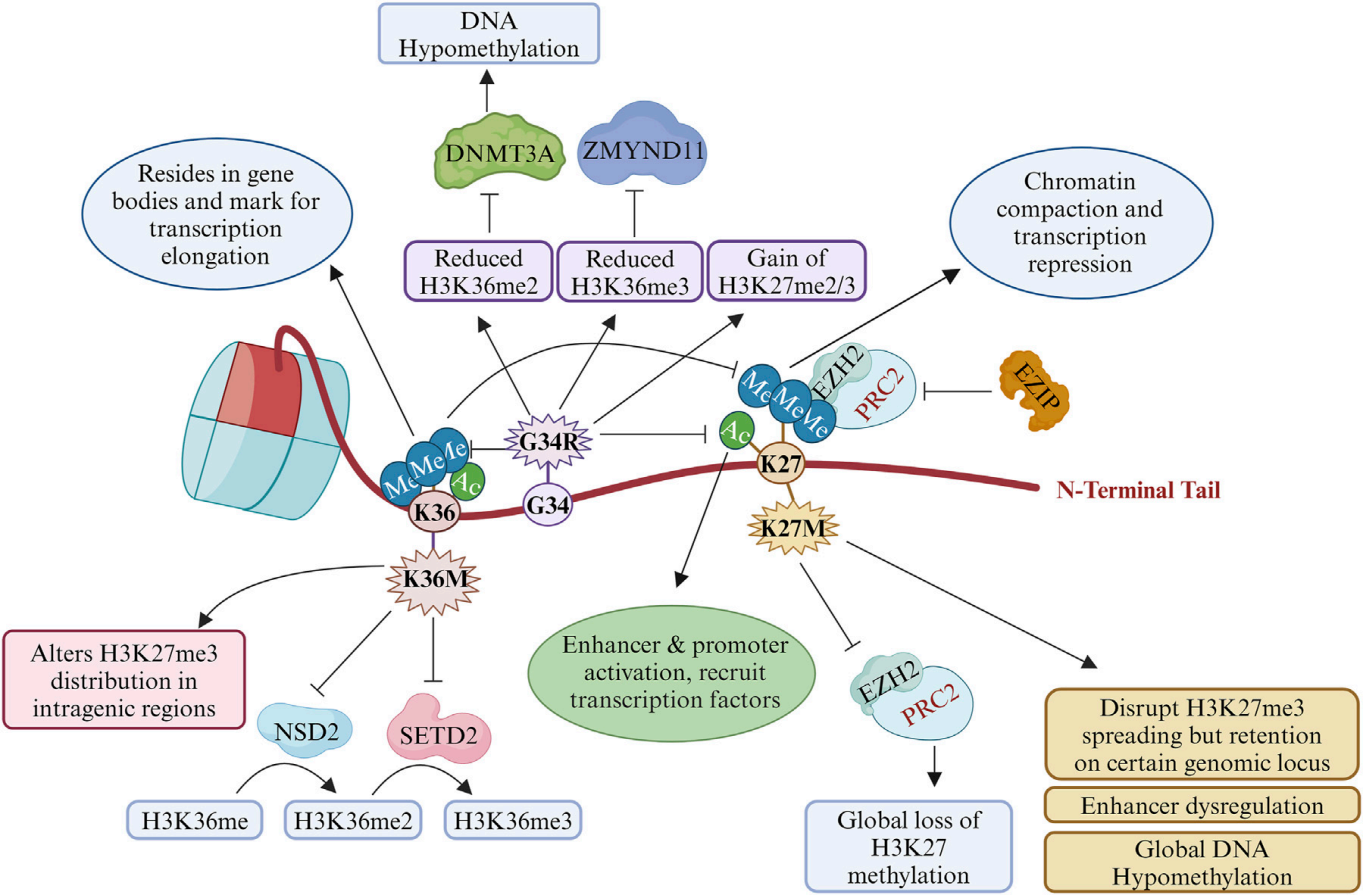
Roles and transcriptional impacts of oncohistones in cancer: post-translational modifications (PTMs) at K27 and K36 by key oncohistone mutations H3K27M, H3G34R, and H3K36M. This illustration describes how these oncohistones alter the bindings of reader, writer, and eraser proteins to promote transcriptional regulation and genomic instability. ZMYND11, zinc finger MYND-type containing 11; NSD2, nuclear receptor-binding SET domain protein 2; DNMT3A, DNA (cytosine-5)-methyltransferase 3 alpha; SETD2, SET domain containing 2; EZH2, enhancer of zeste homologue 2; PRC2, polycomb repressive complex 2; EZIP, EZH2 interaction protein (created with BioRender.com, accessed on 9 June 2024).

The histone H3K27 undergoes methylation and acetylation depending on the context of the developmental signals through a complex interplay of chromatin modifiers, such as methyltransferases, demethylases, acetyltransferases, and deacetylases. H3K27 methylation is regulated by the polycomb repressive complex 2 (PRC2) and recruits PRC1 for monoubiquitination of histone H2A at lysine-119 (H2AK119ub) (Blackledge and Klose, 2021). Their interplay regulates the development of mammalian cells through the regulation of chromatin structure–function relationships (Blackledge and Klose, 2021). Only one H3K27M mutant histone H3 is present along with a WT copy of histone H3; moreover, H3.3K27M inhibits the catalytic subunit of PRC2, namely lysine methyltransferase EZH2 (enhancer of zeste 2, KMT6), and causes global reduction of repressive histone H3K27 trimethylation accompanied by enrichment of H3K27 trimethylation and EZH2 itself at a certain genomic locus (Lewis et al., 2013; Venneti et al., 2013; Chan et al., 2013; Harutyunyan et al., 2019) (Figure 2). The H3K27M mutant binds tightly with EZH2 and blocks its methyltransferase activities as well as genome-wide deposition of H3K27me2 and H3K27me3 (Lewis et al., 2013; Harutyunyan et al., 2019; Justin et al., 2016). However, the EZH2 expression remains unaltered (Venneti et al., 2013). Another model proposes that H3K27M disrupts the auto-methylation of the PRC2 subunits (EZH2 and SUZ12) (Lee et al., 2019). H3.3K27M patient cells show reduced dimethylation and trimethylation of H3K27 globally; however, EZH2 with these PTMs are localised on the cancer-associated genes, suggesting additional mechanisms of action of the H3K27M mutant histone (Chan et al., 2013; Bender et al., 2013). Interestingly, analysis of live-cell single-molecular dynamics of PRC2 suggests that H3.3K27M delays the chromatin residence time and target search time of EZH2 (Tatavosian et al., 2018).

H3.3 in cooperation with acetyltransferase (p300) is required for H3K27 acetylation (H3K27Ac), which is a mark of an active promoter and enhancer (Choi et al., 2024). H3K27Ac co-localises with H3.3K27M and bromodomain proteins at the actively transcribed genes, which excludes PRC2 from the H3K27M-occupied regions (Piunti et al., 2017). H3.3K27M mutants mainly inhibit the spread the of H3K27me3 marks, provided that PRC2 deposition and propagation remain the same (Harutyunyan et al., 2019; Chen et al., 2020a) (Figure 2). This results in dysregulation of the super-enhancers of specific gene clusters, such as the NOTCH pathway genes (Chen et al., 2020a).

#### 3.2.2 H3K27M and H3G34R mutants both impact DNA methylation

The biological context and dynamic interactions between H3K27me3 and DNA methylation regulate the chromatin structure for transcription and other DNA-templated processes such as DNA repair. H3.3K27M and H3.3G34R both reduce the global DNA methylation levels in K27M in pHGGs (Sturm et al., 2012; Bender et al., 2013; Kfoury-Beaumont et al., 2022) (Figure 2). G34 and K27 appear as separate entities in a DNA-methylation-based classification of CNS tumours (Capper et al., 2018; Louis et al., 2021). Since DNA methylation at specific genes regulates stem cell proliferation and stem cell properties, it is not surprising that aberrant expressions of gene patterns are involved in stem cell regulation, differentiation, and tumorigenesis owing to the dominant negative activity of H3K27M expression (Kfoury-Beaumont et al., 2022). To identify the epigenomic factors and other proteins in an unbiased manner, Siddaway et al. (2022) identified the transcription factors, H3K9 methyltransferases, and DNA repair proteins along with many published chromatin modifiers, such as the PRC2 component. Thus, H3K27M alters the chromatin states and transcriptional outputs by changing the epigenomic factor recruitment to profoundly impact tumorigenesis.

#### 3.2.3 H3G34R/V and H3K36M mutations alter the PTMs at H3K36 methylation

H3K36 methylation is crucial for controlling gene transcriptions, and its perturbation will contribute to cancer development. Adult secondary glioblastoma multiforme (GBM) harbours isocitrate dehydrogenase 1/2 (IDH1/2) mutations (about 98%), which are rarely found in childhood GBM, and also regulate methylation at the H3K27 and H3K36 residues (Sturm et al., 2012). This section highlights how H3K36 methylation is perturbed in paediatric CNS tumours as well as soft-tissue bone tumours.

H3G34 lies close to the H3K36 residue that can undergo methylation as well as acetylation and is implicated in several DNA-templated processes such as transcription, dosage compensation, DNA replication, and DNA damage repair (Kallappagoudar et al., 2015; Carpenter, 2012). Histone methylations occur at the arginine or lysine residues, with H3K36 being methylated in three forms. The yeast enzyme SET2, which is a homologue of the human SETD2, generates H3K36 me1/me2/me3 methylation, but separate enzymes such as MMSET or NSD1 or NSD2 (Wolf–Hirschhorn syndrome candidate 1, WHSC1) are responsible for monomethylation and dimethylation in humans while SETD2 undergoes trimethylation (Kallappagoudar et al., 2015; Carpenter, 2012; Sharda and Humphrey, 2022) (Figure 2). H3.3G34R/V blocks the activity of SETD2, which is itself mutated in 15% of pHGGs (Kallappagoudar et al., 2015; Lewis et al., 2013; Huang et al., 2020; Yadav et al., 2017; Fontebasso et al., 2013). Accordingly, each G34 mutant has a differential impact on H3.3K36me2/3 *in cis* (Lewis et al., 2013; Yadav et al., 2017; Khazaei et al., 2023; Lowe et al., 2021; Shi et al., 2018) (Figure 2). Fang et al. (2018) identified a H3 ‘G33-G34’ motif that serves as a docking site for the SETD2 enzyme. Furthermore, in GCTBs, H3.3G34L/W mutants inhibit SETD2 enzymatic activity like the H3.3G34R/V mutants (Shi et al., 2018). The level of H3K36ac also depends on the amino acid substitution at the H3G34 residue (Yadav et al., 2017; Lowe et al., 2021). Therefore, characteristics of amino acid substitution at H3G34 regulate the PTMs at the H3K36 residue.

H3K36me3 is a highly enriched mark on the gene bodies of actively transcribed genes, which are involved in transcriptional elongation and DNA repair in the transcriptionally active regions (Bannister et al., 2005). Reduced H3K36 methylation causes an aberrant gain of H3K27me2/3 and loss of H3K27ac on a gene with SETD2 activity (Jain et al., 2020). G34R cause hypo DNA methylation like H3K27M through reduced recruitment of the decreased DNA methyltransferase DNMT3A by impaired binding with H3K36me2 (Khazaei et al., 2023). H3.3G34R and H3.3K27M mutations modulate the recruitment of H3.3 at the transcriptional active sites (Newhart et al., 2013). On the other hand, the transcriptional repressor ZMYND11 interacts with H3K36 methylation to regulate gene expression while the H3.3G34R mutant abrogates the binding of ZMYND11 (Bressan et al., 2021) (Figure 2). Furthermore, the H3.3G34V mutation prevents ZMYND11 binding to the H3.3K36me3 peptide (Wen et al., 2014). H3.3G34R reduces H3K9me3 and H3K36me3 by inhibiting the enzymatic activities of the KDM4 family of K9/K36 demethylase-like adult counterparts, where the IDH1/2 mutants inhibit KDM4 (Voon et al., 2018). H3K9 methylation and heterochromatin are intact in the H3G34R mutant fission yeast cells (Yadav et al., 2017). These studies implicate the effects of G34 substitution on H3K27 methylation, DNA methylation, H3K36 methylation, and enhancers of the cancer-associated or developmental controlling genes.

H3.3K36M mutant proteins cause global reductions of H3K36 methylation in human chondroblastoma by inhibiting two H3K36 methyltransferases, i.e., NSD2 and SETD2 (Fang et al., 2016). Similar to the H3.3K27M mutants, the H3K36M mutant nucleosomes inhibit H3K36 methyltransferase enzymatic activities, resulting in the loss of H3K36 trimethylation (Lu et al., 2016; Yang et al., 2016) (Figure 2). Furthermore, H3K36M inhibits NSD1, NSD2, and H3K36me2 status phenocopy of the genetic deletions of these methyltransferases (Rajagopalan et al., 2021). However, the GCTB G34 mutations (H3.3G34L/W) reduce H3K36 methylation *in cis* (Shi et al., 2018; Yang et al., 2016). H3.3G34W mutation increases the splicing alterations in GCTBs by interacting with several splicing factors (significant interactor-trans-acting splicing factor hnRNPA1L2) (Lee et al., 2024).

#### 3.2.4 Does the H3K27M mutant alone cause tumorigenesis?

In DIPGs, the H3.3K27M mutation occurs concurrently with p53 mutations and platelet-derived growth factor receptor α polypeptide (PDGFRA) amplification (Khuong-Quang et al., 2012; Sturm et al., 2012; Jones and Baker, 2014; Funato et al., 2014). Expression of H3.3K27M in the neural progenitor cells (NPCs) derived from human embryonic stem cells increases its cellular proliferation (Funato et al., 2014). Furthermore, H3.3K27M with overexpression of the constitutively active PDGFRA mutant and knockdown of p53 has increased cell proliferation, transformation, and tumorigenicity for NPCs (Funato et al., 2014). Single-cell sequencing of the H3.3K27M gliomas suggests that their cell origins resemble oligodendrocyte precursor cells and lack differentiated malignant cells (Filbin et al., 2018). Thus, the development of preclinical glioma models of H3K27M requires an additional mutation in p53 as well as others like PDGF and ACVR1, as reviewed here (Sahu and Lu, 2022). Knockdown of H3.3K27M in DIPG xenografts restores H3K27me3 and inhibits tumour growth. It was found that the loss of H3K27me3 reduces the differentiation of NPCs by regulating the poised promoter status of the cancer-associated genes (Silveira et al., 2019). In *C. elegans,* modelling of H3.3K27M resulted in alteration of H3K27me3 that produces ectopic DNA replication and cell cycle progression (Delaney et al., 2019). Local inhibition of the pre-existing H3K27me3 by H3.3K27M upregulated the Jun amino-terminal kinase (JNK) in germ cells, which may be used as targets for the tumour-derived H3.3K27M cells (Delaney et al., 2019). The yeast system lacks PRC2 and DNA methylation, so *C. elegans* and *Drosophila* may be used as model systems to study H3K27M mutations (Zhang et al., 2023; Chaouch and Lasko, 2021).

#### 3.2.5 Do H3G34R/V and H3K36M mutations cause tumorigenesis?

Engineering the H3.3G34R mutation in human astrocytes showed increased proliferation compared to the WT astrocytes (Chen et al., 2020a). H3.3G34R/V/W knock-in mice show distinct developmental defects, and modelling these mutations in a fission yeast system causes differential genomic instabilities, suggesting that each substitution of the G34 residue produces unique phenotypes (Khazaei et al., 2023; Lowe et al., 2021). This is further illustrated by the role of H3.3K36M in chondrocytes, which exhibit increased colony formation, blocked apoptosis, and differentiation (Fang et al., 2016), such as the differentiation of the mesenchymal progenitor cells. H3.3K36M impacts chondrocyte differentiation and limb development but no tumour development has been recorded in this knock-in mouse model, suggesting additional requirements for tumour development (Abe et al., 2021). However, undifferentiated sarcomas are produced by the H3K36M mutant, suggesting tissue-specific functions of the oncohistone (Lu et al., 2016). H3K36 methylation also alters the genome-wide gain in H3K27 methylation in the H3.3K36M mutant, which redistributes PRC1 and hence de-represses the genes responsible for mesenchymal differentiation in the H3.3K36M mutant cells (Lu et al., 2016). Thus, it is crucial to delineate the individual roles of H3K36me2 and H3K36me3 in H3K36M-driven oncogenesis (Rajagopalan et al., 2021). H3.3K36M alters H3K27me3 distribution through the global loss of H3K36me2 (Abe et al., 2021).

In pHGGs, H3.3G34R/V mutations are accompanied by tumour protein p53 (TP53) loss and PDGFRA amplification (Mackay et al., 2017; Chen et al., 2020b). Furthermore, H3.3G34R/V HGGs are similar to their adult counterparts because both tumours have mutations in the chromatin remodelling protein (ATRX) and TP53 (Schwartzentruber et al., 2012; Chen et al., 2020b; Liu et al., 2012; Korshunov et al., 2016). However, H3.3G34W mutants are found only in mesenchymal tissues (Khazaei et al., 2023; Jain et al., 2020; Chen et al., 2020b). 

H3.3G34R/V inhibits neuronal differentiation and contributes to tumorigenesis through altered gene expressions (Chen et al., 2020b). In glioblastomas, G34 mutations upregulate the oncogene MYCN (Bjerke et al., 2013). These studies highlight the direct impacts of histone mutations on PTMs, which regulate the transcriptional profiles of tumour cells. However, these PTMs also regulate the DDR pathways in cells, so we discuss how histone mutations play crucial roles in genomic instabilities by modulating the epigenome through altered PTM profiles in the tumour cells.

## 4 Histone H3.3 and its PTMs at K27 and K36 contribute to DDR in several cell types

Histones can be classified as canonical (H3.1 and H3.2) or replication-dependent histones and non-canonical (H3.3) or replication-independent histone variants depending on their requirements during DNA replication (Talbert and Henikoff, 2010). A countable number of amino acid differences between these variants make them distinguishable in terms of chaperone binding, chromatin localisations, and other functions (Kallappagoudar et al., 2015; Ferrand et al., 2020; Szenker et al., 2011). The protein H3.3 plays an extensive role in regulating chromatin structures and cellular differentiation (Cohen and Meshorer, 2024; Shi et al., 2017). During DNA damage signalling, chromatin remodelling occurs through deposition of H3.3 and is critically regulated by ATRX and the switch/sucrose non-fermentable (SWI/SNF) family chromatin remodeller protein CHD1 to regulate genomic stability during transcription as well as replication (Choi et al., 2024). For additional details, readers are referred to reviews on chaperones and other complexes (Choi et al., 2024; Szenker et al., 2011). Extensive deposition of the histone variant H3.3 across the chromosomal domain has been reviewed through different chaperone binding activities that regulate the maintenance of chromosomal integrity and DDR, including the chromatin dynamics during DNA damage (Ferrand et al., 2020). Mouse models suggest that during mammalian development, the H3.3 null mutant causes defects in the heterochromatic structures and genome integrity (Jang et al., 2015).

**FIGURE 3:**
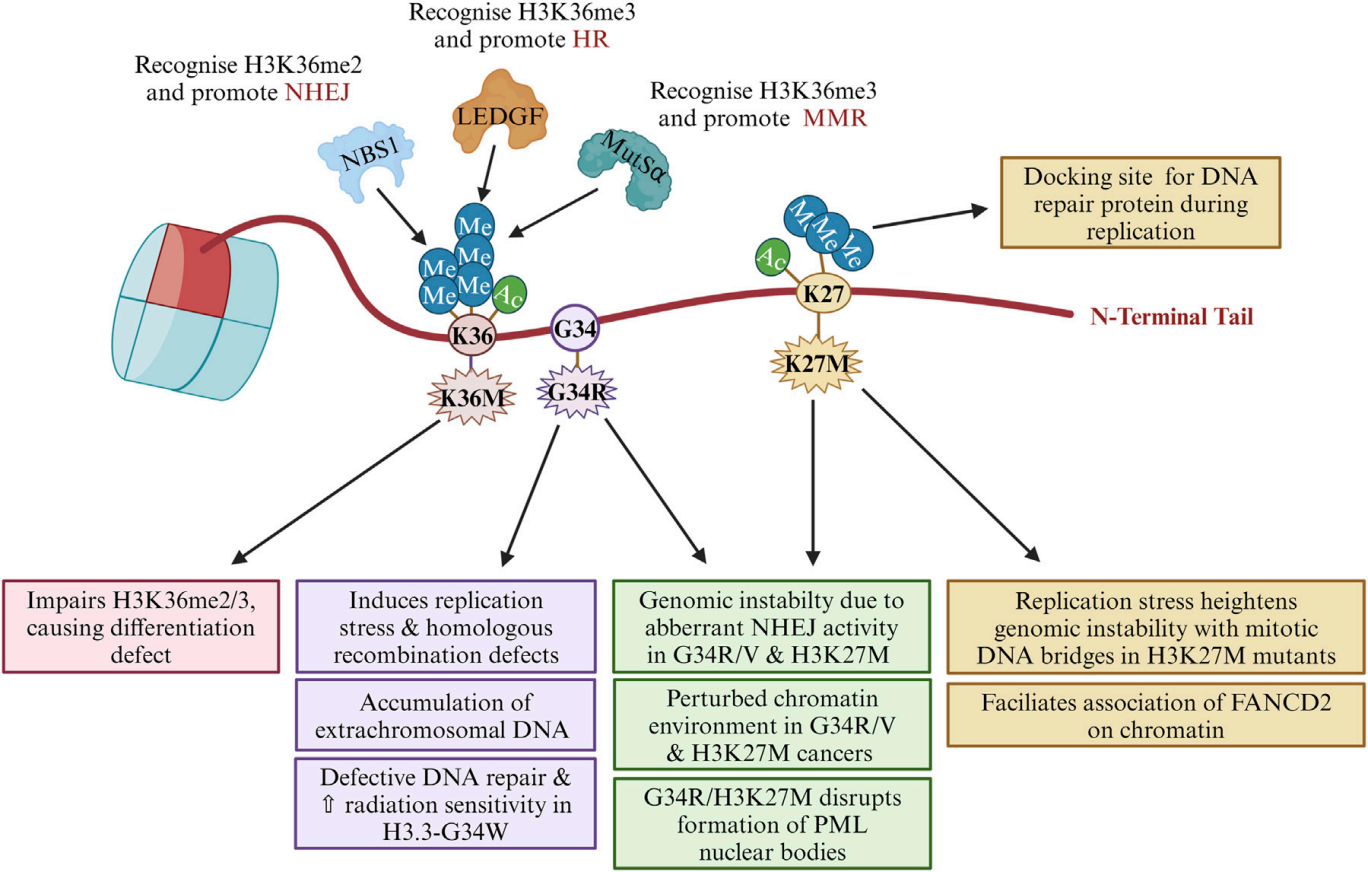
Oncohistone descriptions in genomic instability: contributions of histone PTMs at H3K27, H3G34, and H3K36 to DNA repair pathway choice. This figure illustrates the roles of the PTMs at histone H3 lysine-27 (H3K27) and lysine-36 (H3K36) in promoting genomic instabilities. Specific PTMs at these residues are depicted, highlighting their influences on the chromatin structure and functions, particularly in the context of DNA damage response (DDR) and replication stress. The schematic representation illustrates how these oncohistone mutations disrupt normal chromatin dynamics and DDR processes, increasing replication stress and genomic instability. FANCD2, Fanconi anaemia complementation group D2; LEDGF, lens epithelium-derived growth factor; NBS1, Nijmegen breakage syndrome 1; MutSα, MutS homologue alpha (MSH2-MSH6 heterodimer) (created with BioRender.com, accessed on 9 June 2024).

When DNA damage occurs during transcription, evidence suggests that the locus must be silenced to avoid conflicts of the transcription machinery with the DNA repair complexes (Campbell et al., 2013). Upon DNA damage and repair, transcription recovery at the locus requires H3.3 deposition (Frey et al., 2014; Adam et al., 2013). H3.3 deposition is also required during the S phase only when cells encounter UV irradiation, suggesting the importance of the histone H3.3 during transcription and replication-associated DNA damage (Frey et al., 2014; Adam et al., 2013). Furthermore, the repair depends upon the DNA damage-associated poly (ADP-ribose) polymerase 1 (PARP1) activity, attracting the chromatin remodelling complex and other gene silencing proteins to shut off nearby gene transcriptions to facilitate DNA repair (Chou et al., 2010; O'Hagan et al., 2011). Alternative to these mechanisms, PARP1 also recruits CHD2 that triggers chromatin relaxation and H3.3 deposition at the DNA damage locus (Dabin et al., 2023; Luijsterburg et al., 2016). Furthermore, EZH2-dependent H3K27me3 marks may act as molecular ‘timers’ of DDR pathways when DNA damage occurs during replication (Ito et al., 2018) (Figure 3). The G34 small residue lies between H3K27 and H3K36 and may control the binding of epigenetic factors for DDR through its impact on the PTMs of K267 and K36.

DNA lesions occur in the context of chromatin, which controls the choice of pathways by which these lesions are repaired (Figure 3). CNS tumours are mostly managed clinically through radiation, which produces DNA double-strand breaks (DSBs). The main repair pathways are DNA non-homologous end-joining (NHEJ) and homologous recombination (HR). Several factors determine the fate of DSB repairs, such as cell cycle stages, chromatin environment surrounding the DNA lesions, availability of repair proteins, compartmentalisation within the nucleus, and transcription status around the lesions (Arnould et al., 2023; Campbell et al., 2013). Furthermore, selective utilisation of NHEJ *versus* HR is evident in nervous system development (Jackson and Bartek, 2009; Orii et al., 2006).

H3K36me3 resides on the gene body and marks the elongation of transcription (Barski et al., 2007). Interestingly, its role in genomic instability and other cellular functions is found to increase each day (Carpenter, 2012). H3K36me3 modulates DNA repairs at the transcriptionally active regions as well as DNA lesions through mismatch repair (MMR) (Sharda and Humphrey, 2022; Fang et al., 2018). H3K36me2 promotes NHEJ, whereas H3K36me3 promotes HR as the PTMs are recognised by Nijmegen breakage syndrome 1(NBS1) and lens epithelium‐derived growth factor (LEDGF) proteins, respectively (Sharda and Humphrey, 2022; Fnu et al., 2011; Pfister et al., 2014) (Figure 3). Depletion of H3K36me3 leads to decreased ataxia-telangiectasia mutated (ATM) and p53 phosphorylations, defective DNA end-resection, impaired damage recruitment of RPA and RAD51, and low HR efficiency; it provides binding sites for the PWWP methyl reader domain of LEDGF, promoting HR repair through interactions with the C-terminal binding protein 1 (CtBP1) interacting protein (CtIP) (Aymard et al., 2014). Overexpression of H3K36me3 demethylase KDM4A reduces HR efficiency in cells. Dimethylation of H3K36 is induced by ionising radiation and accumulates around the DSBs, leading to increased accumulation of NHEJ factors (Pfister et al., 2014). Klein et al. (2018) found that the common cancer-related substitution of H3K36 to methionine disrupts the H3K36me-writing enzymes and H3K36me-specific readers, potentially leading to oncogenic effects.

## 5 H3K27M, H3G34R/V, and H3K36M roles in genomic instability or DDR

Loss of heterozygosity of the DNA repair pathway genes and a slight gain in PARP1 expression were found in low samples of DIPGs (Zarghooni et al., 2010). Receptor tyrosine kinase (RTK) inhibitors are often targeted in CNS tumours and other cancers. However, recent studies suggest that signalling events can modulate, stimulate, or inhibit HR or the NHEJ repair pathways (Chabot et al., 2021; Liu et al., 2023). Investigators have found that DDR mechanisms within tumour cells are dysregulated, which can cause tumorigenesis or can be exploited as chemotherapeutic agents in cancer treatment (Rominiyi and Collis, 2022). Therefore, it is essential to develop a protocol to measure the efficiency of the DNA repair pathways in cancer therapy. For example, the recombination proficiency score (RPS) is calculated based on the expressions of the DNA repair pathway genes (Rif1, PARI, RAD51, and Ku80) to determine the fate of chemotherapy (Pitroda et al., 2014). In the next section, we examine the roles of H3K27M, H3G34R/V, and H3K36M in literature for genomic instability or DDR; this helps identify many combined strategies to target both the DDR and RTK signalling pathways (Liu et al., 2023).

### 5.1 Chromosomal abnormalities in CNS tumours

Genomic instability plays a pivotal role in tumorigenesis, contributing to the development and progression of cancer through various mechanisms. Genomic instability essentially refers to the fact that cancer cells exhibit higher rates of genetic alterations, such as copy number alterations (CNAs), chromosomal rearrangements, and mutations, than normal cells, as discussed for CNS tumours in the previous section. This instability results from various causes, including defects in the DNA repair mechanisms, replication stress, and abnormal telomere maintenance pathways; it is a hallmark of cancer and a major cause of tumorigenesis (Hanahan, 2022).

Comparisons of paediatric and adult glioblastomas show that (a) frequent gains of chromosome 1q were 30% and 9%, respectively; (b) chromosome seven gains were 13% and 74%, respectively; (c) chromosome 10q losses were 35% and 80%, respectively (Paugh et al., 2010). Radio resistance is commonly observed in DMGs (Liu et al., 2023). On the contrary, radiation-induced tumours show significant increases in PDGFRA amplification and 1q gains in childhood gliomagenesis (Paugh et al., 2010). Adult CNS tumours have high CDK6 amplification, 10q loss, and 17q gain, whereas paediatric cases have a high frequency and high specificity of 3q and 4q losses across MYC/MYCN oncogene amplification, suggesting variations in the chromosomal abnormalities between adult and paediatric CNS tumours (Korshunov et al., 2016; Liu et al., 2023; Korshunov et al., 2010)**.**

### 5.2 Contributions of H3K27M, H3G34R/V, and H3K36M mutations to DDR pathways

During tumour development and ongoing treatment, several processes regulate DNA repair and cell cycle gene expressions. High CNAs are reported in H3.3 mutant gliomas, and mitotic abnormalities such as mitotic bulky and ultrafine DNA bridges were observed in an inducible H3.3K27M cell culture model (Bockaj et al., 2021). This is similar to the replicative stress mechanism reported in a fission yeast model for H3G34R mutation (Yadav et al., 2017). Accumulation of extrachromosomal DNA was observed in the H3.3G34R model mouse and H3.3G34R-harbouring human pHGG cells because of downregulation of the DNA repair pathway genes (Haase et al., 2022). Consistent with this, the H3.3G34R fission yeast cells show lagging chromosomes and are sensitive to replication-stress-specific DNA-damaging drugs (Yadav et al., 2017; Lowe et al., 2021). Thus, it is evident that H3.3 mutant cells display increased genomic instability phenotypes owing to compromised DDR responses. It may also be speculated that H3.3G34R and IDH1/2 mutations co-operate with ATRX-mutated glioblastomas, leading to alternative lengthening of the telomeres, which is a process that is sometimes regulated through the HR pathways in the absence of or compromised functions of telomerase genes (Udugama et al., 2021). These studies suggest that DNA damage occurs through replication in the H3G34R/H3K27M cells.

H3.3K27M/G34R mutations disrupt the formation of promyelocytic leukaemia (PML) nuclear bodies that are the main drivers of leukaemia in the blood (Voon et al., 2023) (Figure 3). This is crucial as the PML nuclear bodies are important regulators of genome maintenance, and their disruption sensitises the H3.3-mutated glioma cells (Voon et al., 2023; Chang et al., 2018). In adult CNS tumours, the IDH1-R132H mutant epigenetically upregulates DDR and also disrupts the formation of PML bodies (Nunez et al., 2019). 

H3.1K27M-engineered human dermal fibroblast cells show reduced foci for 53BP1, an NHEJ protein (Zhang et al., 2018). Consistent with this, increased rates of genomic insertions or deletions and copy number variations occur through p53-dependent apoptosis in these cells (Zhang et al., 2018). Furthermore, hypo-methylation on H3K27 decreases the NHEJ efficiency and facilitates association of Fanconi anaemia complementation group D2 (FANCD2) on the chromatin, which is a central player in the choice of DNA repair pathway (Zhang et al., 2018; Cohn and D'Andrea, 2008) (Figure 3).

Interestingly, H3.3G34W shows DSB repair defects in bone tumours that are sensitive to ionising radiation (IR). It was reported that the enhanced interactions of H3.3G34W with damaged nucleosomes led to dysregulated interactions with the NHEJ key effectors, such as KU70/80 (Mancarella et al., 2024). 

H3.3G34R/V/D mutants have shown reduced interactions with the MMR protein MutS homologue α (MutSα; an MSH2-MSH6 heterodimer) and a mutator phenotype similar to that of MMR-defective cells (Fang et al., 2018) (Figure 3). This is due to the reduced recruitment of MutSα by the reduction of H3K36me3. However, further studies are required to highlight the importance of MMR pathways in the context of tumorigenesis (Dabin et al., 2023).

H3K36M oncohistone mutation inhibits SETD2 methyltransferase activity, with S-adenosylmethionine (SAM) indirectly affecting the interactions and maintaining the proper fold state in the SETD2-H3K36M-SAM complex structure (Zhang et al., 2017). Although H3K27M and H3G34R/V mutations have been extensively studied for their roles in paediatric gliomas and other cancers, H3K36M is less explored yet equally critical. Unlike H3K27M, which is known for its repressive effects on the polycomb group proteins, H3K36M primarily affects the methylation landscape associated with actively transcribed genes.

## 6 Can genomic instabilities and additional genetic requirements of oncohistone-containing cancer cells be exploited as a therapeutic regimen?

**FIGURE 4:**
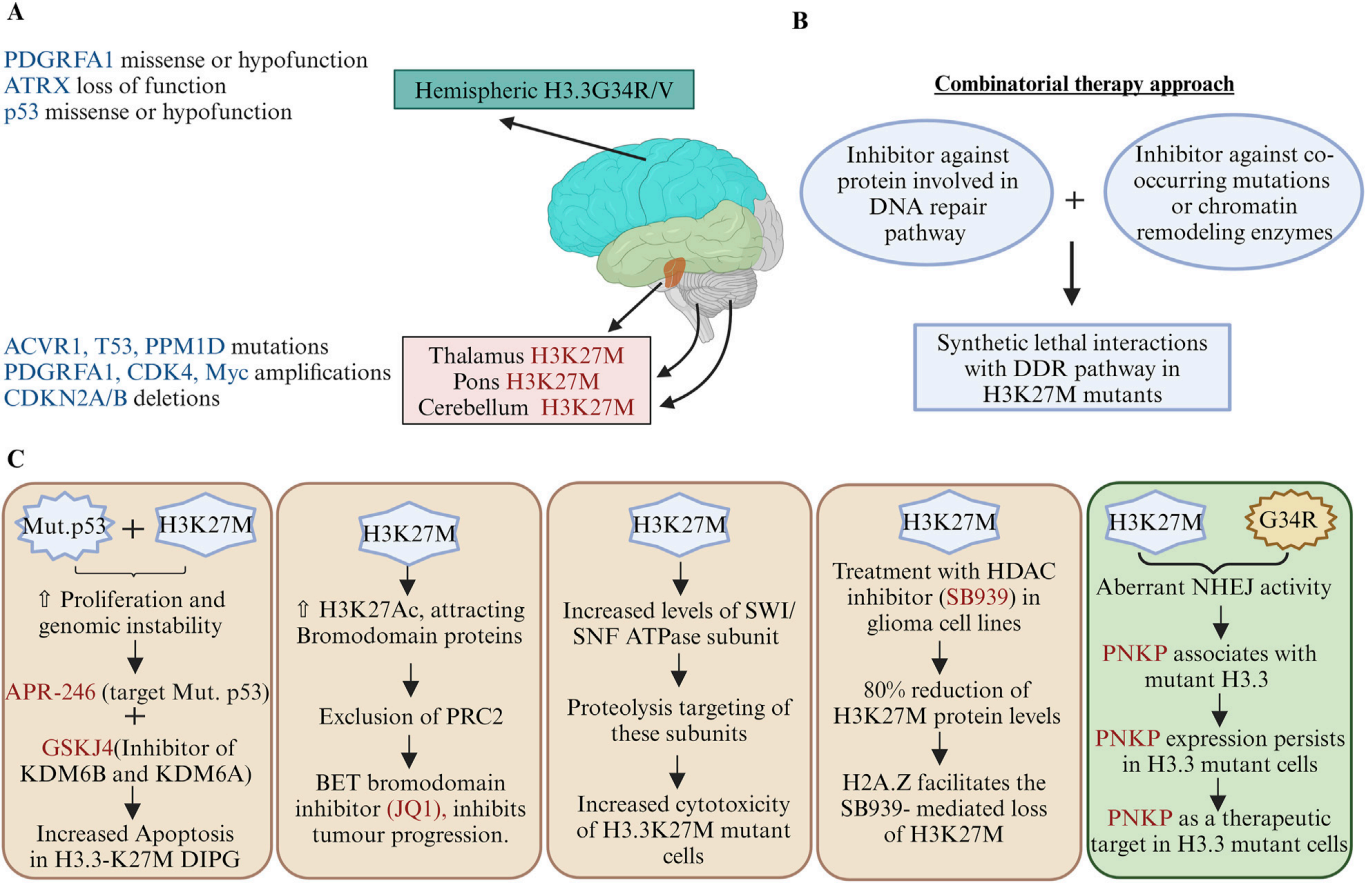
Therapeutic strategies targeting oncohistone mutations: **(A)** Anatomical distribution and genetic landscape of oncohistone mutations; the diagram illustrates the distribution of histone mutations H3K27M and G34R/V across different brain regions, highlighting their associations with various genetic alterations. **(B)** Synthetic lethal interactions with the DDR pathway; a combination of inhibitors against DNA repair pathway proteins and chromatin remodelling enzymes may be exploited to target cancer cells with histone mutations. **(C)** Therapeutic strategies targeting oncohistone mutations; mechanistic impacts of H3K27M and G34R/V mutations on cancer cell survival and their genetic requirements for other cancer-associated genes. Few illustrations depict how a potential therapeutic target can be designed to reduce the growth of H3.3 mutant cancer cells. For example, the combination of APR-246 and GSKJ4 enhances apoptosis in H3.3K27M DIPGs, addressing how co-mutations of p53 and H3K27M-associated dysregulations in the epigenomic profile may be exploited to identify novel and precise cancer treatments. PDGFRA1, platelet-derived growth factor receptor alpha 1; ATRX, alpha thalassemia retardation X-linked; ACVR1, activin A receptor type 1; p53, tumour protein 53; PPMID, protein phosphatase Mg^2+^/Mn^2+^-dependent 1D; CDK4, cyclin-dependent kinase 4; CDKN2A/B, cyclin-dependent kinase inhibitor 2A/2B; SWI/SNF, switch/sucrose non-fermentable; PNKP, polynucleotide kinase 3′-phosphatase (created with BioRender.com, accessed on 9 June 2024).

pHGG histone mutants are a double-edged sword as these cancer cells are proliferative as well as have genomic instability phenotypes, as discussed in Sections 3 and 5. Therefore, finding synthetic lethal interactions between histone mutants with DDR pathway genes or co-occurring mutational genes may offer promising therapeutic approaches for cancers with histone mutations (Figure 4). Screening has been used to identify vulnerabilities in the DDR-deficient cells, which can be targeted with specific inhibitors or combination therapies to selectively eliminate cancer cells while sparing normal cells. Blum (1950) hypothesised that successive doses of UV radiation increases the rate of cellular proliferation with unclear mechanisms. Genomic instability contributes to cancer progression; however, increased proliferation may pose challenges to accurately repair the error rates in replication due to various oncogenic replication stresses. Activated oncogenes produce DNA DSBs due to stalling and collapse of the DNA replication forks during continuous cell proliferation, and TP53 acts as the guardian of genomic stability (Halazonetis et al., 2008). However, mutant p53 is oncogenic through inactivation of the DNA damage sensor protein ATM activation or promotion of the chromatin association and nuclear activity of PARP1 for alternative means to counter genomic instabilities in cancer cells (Song et al., 2007; Polotskaia et al., 2015). Since the H3.3K27M mutation occurs concurrently with a mutation in p53, the APR-246 drug (target mutant p53) produces oxidative stress in H3.3K27M DIPGs (Nikolaev et al., 2020) (Figure 4). Therefore, the context of activated mutant oncogenes or mutant tumour-suppressor proteins as targets is important.

Although DNA repair deficiencies can cause cancer evolution, PARP1 inhibitors can be used as treatment modalities in DNA-damage-compromised cells (Rominiyi and Collis, 2022; Volkova et al., 2020). DNA damage contributes to tumorigenesis by providing mutational clonality so that the normal cells may promote cancer cells; however, excessive DNA damage in cancer cells can be exploited for chemotherapy. Lin et al. (2019) proposed a mechanism-based drug design strategy for targeting drug-resistant gliomas; this strategy is promising for overcoming drug resistance and improving glioma treatment outcomes.

Giacomini et al. (2024) reported a novel mechanism driving pHGGs and highlighted aberrant DNA repair as a key contributor to tumour development. Mutations in the histone H3.3, particularly at K27M and G34R, promote genome instabilities by enhancing the NHEJ repair of replication-associated damage. They suggested that polynucleotide kinase 3′-phosphatase (PNKP) is a critical mediator of this aberrant repair process, showing increased association with mutant H3.3; hence, targeting PNKP is a promising therapeutic strategy for gliomas and other cancers with these mutations (Figure 4). Although H3K27 methylation is impaired, PRC2 is necessary for the proliferation of H3.3K27M-expressing tumours (Mohammad et al., 2017). EZH2 inhibitors abrogate the cell growths of these tumours through upregulation of the tumour suppressor protein p16INK4A (Mohammad et al., 2017). Bromodomain protein inhibitors (JQ1) can reduce DIPG cell proliferations by enhancing their terminal neuronal differentiation (Piunti et al., 2017) (Figure 4).

Components of the chromatin remodelling SWI/SNF complex proteins are upregulated in H3.3K27M gliomas, and their degradation leads to reduced viability of H3.3K27M cells (Mota et al., 2023). GSK-J4 is a Jumonji family histone demethylase (JMJD3) inhibitor. H3K27 methylation increases in H3.3K27M cells upon treatment with GSK-J4 and has antitumour activities for cells harbouring H3.3K27M *in vitro* as well as *in vivo* (Hashizume et al., 2014). The mutant-p53-targeting drug APR-246 produces oxidative stress, and combining it with GSKJ4 increases apoptosis in H3.3K27M DIPGs (Nikolaev et al., 2020) (Figure 4). Histone deacetylase (HDAC) inhibitors like pracinostat/SB939, panobinostat, vorinostat, and entinostat destabilise the H3.3K27M protein in multiple glioma cell lines (Leszczynska et al., 2024). Mechanistically, co-occurrence of the H2A.Z histone with H3.3K27M nucleosome facilitates loss of H3.3K27M but is inhibited by chloroquine, which is an inhibitor of autophagosome-lysosomal degradation and a DNA-intercalating agent (Leszczynska et al., 2024).

In cancer cells, DNA repair and/or DDR factors functionally interact with chromatin to orchestrate the DNA repair processes by endogenous or exogenous DNA lesions generated through tumorigenic progression or DNA damaging agents used during chemotherapy. Combination therapies are often dependent upon the interactions of the genes with the drugs (Lin et al., 2019). Synthetic lethal interactions with DDR pathways may also be used as a therapeutic approach. A combination of radiotherapy and DDR inhibitors (DDRi) has been shown to reduce tumour growth in H3.3G34R pHGG-bearing mice (Haase et al., 2022) (Figure 4).

The H3K27M and H3G34R/V mutations disrupt the chromatin structure and functions, resulting in defects in the recruitment of DNA repair proteins, thereby impairing the DNA repair efficiency kinetics and increasing genomic instability. All these events promote tumour progression with distinct molecular mechanisms. However, further investigations into the specific molecular mechanisms underlying these dysregulations are required to identify potential therapeutic targets. Consequently, understanding the DNA repair mechanisms underlying these mutations as well as their implications in targeted therapeutic strategies are critical for advancing precision medicine approaches to glioma treatment.

## 7 Histones H4, H2A, H2B, and H1 are mutated in various cancers

Although this review primarily addresses histone H3 mutations and their implications to the DDR pathways and genomic instabilities, mutations in other canonical core histones, such as H2A, H2B, and linker histone H1, are significantly prevalent across various cancers. Existing literature provides limited insights into how the mutations in these histone variants contribute to the DDR pathways and genomic instabilities. Nevertheless, such mutations are known to disrupt the nucleosomal structures and lead to deregulation of gene expressions.

One such mutation is the H2BE76K mutation found in bladder and head and neck cancers, which tends to form stable dimers with H2A but disrupt H2B-H4 interactions and prevent stable histone octamer formation (Espinoza Pereira et al., 2023; Arimura et al., 2018). Genes upregulated in the H2BE76K mutant cells are involved in cell adhesion and proliferation. In the breast cancer cell line, the transcription of ADAM19 (a disintegrin and metalloproteinase-domain-containing protein 19), a cancer-associated gene, increases by H2BE76K mutation through facilitation of transcription elongation processes (Kang et al., 2021). Unlike other histone mutants, the H2BE76K variant does not impact the global levels of histone PTMs but has been observed to increase chromatin accessibility at the promoters and enhancers via nucleosomal dysfunctions (Kang et al., 2021).

Other examples include the H2BG52D and H2BP102L oncohistones, which affect HR repair by impairing histone eviction and RAD51 recruitment; the heterozygotes for these mutants also exhibit increased genotoxic sensitivity and concomitant reductions in H2B ubiquitination (H2Bub) *in cis* (Qin et al., 2024). The H2BG52D mutation identified in pancreatic cancer and other malignancies impairs DNA–histone interactions, leading to decreased nucleosome stability and disrupted gene regulation. Although H2BG52D does not affect cell proliferation, it significantly enhances cell migration and accelerates wound closure in assays, indicating its role in promoting cancer progression (Wan et al., 2020). Missense mutations in the histone H2A gene account for approximately 20% of all histones missense mutations observed in cancers. The most common H2A mutated residue E121 decreases nucleosome sliding, and another commonly mutated residue R29 increases nucleosome sliding while decreasing stability and enhancing dimer exchange (Bagert et al., 2021).

Other H2A (sH2A) histone variants, which are predominantly expressed in the testes during spermatogenesis in placental mammals, are essential for normal testicular functions (Molaro et al., 2018). These variants are known to destabilise nucleosomes and modulate alternative splicing, as evidenced by studies in germline mutant mice. Additionally, research has demonstrated that peptides derived from the sH2A.B variant can interact with human leucocyte antigen (HLA) molecules, suggesting a potential role for sH2A.B in immune evasion (Lundegaard et al., 2008). Aberrant expressions of the sH2A variants have been observed in several cancers, including diffuse large B-cell lymphoma and Hodgkin’s lymphoma. These dysregulated expressions of the sH2A variants contribute to the oncogenic phenotypes seen in these malignancies (Chew et al., 2021).

The linker histone H1 that is essential for chromatin condensation and gene repression is often mutated in mature B-cell neoplasms and is recognised in literature for its tumour suppressor role. Depletion of H1 cause reduction of H3K27 methylation but increase in H3K36 methylation, altered chromatin compartmentalisation, and enhanced chromatin interactions (Willcockson et al., 2021). Among the cancer-associated mutations, S101F in H1 results in a loss-of-function phenotype, disrupting the interaction between H1 and DNA. Pan-cancer analyses also show mutations at G102 and S103 that may impair DNA binding. Mutant H1 demonstrates accelerated dissociation and diminished chromatin association, compromising chromatin binding and compaction (Yusufova et al., 2021).

Several histone H4 mutations such as R3C, L49F, S1C, and K79N have been reported in various cancers; although the precise roles of these mutations remain unclear, their high frequency of occurrence in cancers suggests their potential roles in oncogenic processes and chromatin dysregulation (Nacev et al., 2019). 

The H4R3C mutation is the most mutated histone H4 residue in cancers and disrupts H4R3me2, which is catalysed by Protein arginine methyltransferase 5 and linked to transcriptional repression at specific genomic loci (Chen et al., 2017). H4R3me2s also serves as a binding site for DNMT3A, promoting DNA methylation (Zhao et al., 2009). Loss of H4R3me2 due to H4R3C mutation leads to reduced DNMT3A binding, decreased DNA methylation, gene activation, and may contribute to oncogenesis.

The H4 H75E mutation in the LRS domain, which engages with H2B, impedes global genomic nucleotide excision repair by disrupting the recruitment of RAD4 to the chromatin. This reduction in DNA repair efficiency occurs without affecting the chromatin structure or accessibility, thereby diminishing the effectiveness of damage recognition (Selvam et al., 2019). Additionally, the residues D68 and R92, which are commonly mutated in cancers, are critical for hydrogen bonding with H2B. Alterations in these residues can likely impair H4-H2B interactions, leading to nucleosomal instabilities (Nacev et al., 2019).

## 8 Conclusion

The contributions of epigenetic mechanisms are crucial for identifying the tumour stages as well as heterogeneities. Vulnerabilities of the chromatin structure can be exploited to target cancer. pHGGs harbour missense mutation of H3.3K27M and H3.3G34R/V, whereas bone tumours have H3.3K36M mutations. Substantial studies have reported that these mutations promote tumorigenesis (known as oncohistones). Selection of tissue-specific cell lines may play significant roles in determining the oncogenic potentials of histone mutations identified upon genome sequencing of cancer cells. Many studies show that the oncogenic potential of a histone mutation depends on co-occurring mutations in other proteins. DDR network perturbation in the H3.3K27M and H3.3G34R/V mutant cancer cells needs further investigation so that we can identify the synthetic lethality between the DNA repair genes and oncohistones. Additionally, histone mutants may perturb chromatin in a context-dependent manner by dysregulating the chromatin modifying complexes. Therefore, one may consider targeting epigenetic complexes to achieve synthetic lethality in tumours harbouring histone mutants. Histones H3K27M and H3K36M are *trans*-acting whereas the H3.3G34 mutants are *cis*-acting, suggesting that cancer-associated histone mutations may differentially modulate PTMs on the histone tails. Furthermore, enrichment of the histone PTMs on the chromatin domains may vary with the nature of the histone mutations, which could regulate gene expression patterns or other DNA-templated processes, such as DNA repairs in oncohistone-containing cancer cells.

The following are some of the major questions that remain unanswered or have partial data to support them:

• Oncogenic histone mutations cause genome instabilities in cancer cells. How do specific repair pathways contribute to the survival of cancer cells in oncohistones?
• Are there any common mechanisms of genome instabilities due to H3.3K27M and H3.3G34R/V histone mutations?
• H3.3K27M and H3.3G34R/V mutant cancer cells have perturbed chromatin environments. Can epigenetic drugs be combined with conventional chemotherapeutics for the treatment of these cancers?

Although we have mainly discussed H3 mutations in this review, other canonical core histones, such as H2A, H2B, and linker histone H1, are also mutated in cancers, and their roles in tumorigenesis constitute active research areas. It is crucial to dissect the functionality of histone mutations to design combination therapies, immunotherapies, or precision medicines in cancer management.

## References

[B1] Abe S. Nagatomo H. Sasaki H. Ishiuchi T. (2021). A histone H3.3K36M mutation in mice causes an imbalance of histone modifications and defects in chondrocyte differentiation. Epigenetics 16 (10), 1123–1134. 10.1080/15592294.2020.1841873 33135541 PMC8510613

[B2] Adam S. Polo S. E. Almouzni G. (2013). Transcription recovery after DNA damage requires chromatin priming by the H3.3 histone chaperone HIRA. Cell 155 (1), 94–106. 10.1016/j.cell.2013.08.029 24074863

[B3] Allis C. D. Jenuwein T. (2016). The molecular hallmarks of epigenetic control. Nat. Rev. Genet. 17 (8), 487–500. 10.1038/nrg.2016.59 27346641

[B4] Amatori S. Tavolaro S. Gambardella S. Fanelli M. (2021). The dark side of histones: genomic organization and role of oncohistones in cancer. Clin. Epigenetics 13 (1), 71. 10.1186/s13148-021-01057-x 33827674 PMC8025322

[B5] Arimura Y. Ikura M. Fujita R. Noda M. Kobayashi W. Horikoshi N. (2018). Cancer-associated mutations of histones H2B, H3.1 and H2A.Z.1 affect the structure and stability of the nucleosome. Nucleic Acids Res. 46 (19), 10007–18. 30053102 10.1093/nar/gky661 PMC6212774

[B6] Arnould C. Rocher V. Saur F. Bader A. S. Muzzopappa F. Collins S. (2023). Chromatin compartmentalization regulates the response to DNA damage. Nature 623 (7985), 183–192. 10.1038/s41586-023-06635-y 37853125 PMC10620078

[B7] Aymard F. Bugler B. Schmidt C. K. Guillou E. Caron P. Briois S. (2014). Transcriptionally active chromatin recruits homologous recombination at DNA double-strand breaks. Nat. Struct. Mol. Biol. 21 (4), 366–374. 10.1038/nsmb.2796 24658350 PMC4300393

[B8] Bagert J. D. Mitchener M. M. Patriotis A. L. Dul B. E. Wojcik F. Nacev B. A. (2021). Oncohistone mutations enhance chromatin remodeling and alter cell fates. Nat. Chem. Biol. 17 (4), 403–411. 10.1038/s41589-021-00738-1 33649601 PMC8174649

[B9] Bannister A. J. Schneider R. Myers F. A. Thorne A. W. Crane-Robinson C. Kouzarides T. (2005). Spatial distribution of di- and tri-methyl lysine 36 of histone H3 at active genes. J. Biol. Chem. 280 (18), 17732–17736. 10.1074/jbc.M500796200 15760899

[B10] Barski A. Cuddapah S. Cui K. Roh T. Y. Schones D. E. Wang Z. (2007). High-resolution profiling of histone methylations in the human genome. Cell 129 (4), 823–837. 10.1016/j.cell.2007.05.009 17512414

[B11] Behjati S. Tarpey P. S. Presneau N. Scheipl S. Pillay N. Van Loo P. (2013). Distinct H3F3A and H3F3B driver mutations define chondroblastoma and giant cell tumor of bone. Nat. Genet. 45 (12), 1479–1482. 10.1038/ng.2814 24162739 PMC3839851

[B12] Bender S. Tang Y. Lindroth A. M. Hovestadt V. Jones D. T. Kool M. (2013). Reduced H3K27me3 and DNA hypomethylation are major drivers of gene expression in K27M mutant pediatric high-grade gliomas. Cancer Cell 24 (5), 660–672. 10.1016/j.ccr.2013.10.006 24183680

[B13] Bjerke L. Mackay A. Nandhabalan M. Burford A. Jury A. Popov S. (2013). Histone H3.3. mutations drive pediatric glioblastoma through upregulation of MYCN. Cancer Discov. 3 (5), 512–519. 10.1158/2159-8290.CD-12-0426 23539269 PMC3763966

[B14] Blackledge N. P. Klose R. J. (2021). The molecular principles of gene regulation by Polycomb repressive complexes. Nat. Rev. Mol. Cell Biol. 22 (12), 815–833. 10.1038/s41580-021-00398-y 34400841 PMC7612013

[B15] Blum H. F. (1950). On the mechanism of cancer induction by ultraviolet radiation. J. Natl. Cancer Inst. 11 (3), 463–495. 14824912

[B16] Bressan R.B. Southgate B. Ferguson K.M. Blin C. Grant V. Alfazema N. (2021). Regional identity of human neural stem cells determines oncogenic responses to histone H3.3 mutants. Cell Stem Cell. 28 (5), 877–93. 33631116 10.1016/j.stem.2021.01.016 PMC8110245

[B17] Bockaj I. Martini T. E. I. de Camargo Magalhaes E. S. Bakker P. L. Meeuwsen-de Boer T. G. J. Armandari I. (2021). The H3.3K27M oncohistone affects replication stress outcome and provokes genomic instability in pediatric glioma. PLoS Genet. 17 (11), e1009868. 10.1371/journal.pgen.1009868 34752469 PMC8604337

[B18] Bonner E. R. Dawood A. Gordish-Dressman H. Eze A. Bhattacharya S. Yadavilli S. (2023). Pan-cancer atlas of somatic core and linker histone mutations. NPJ Genom Med. 8 (1), 23. 10.1038/s41525-023-00367-8 37640703 PMC10462747

[B19] Campbell S. Ismail I. H. Young L. C. Poirier G. G. Hendzel M. J. (2013). Polycomb repressive complex 2 contributes to DNA double-strand break repair. Cell Cycle 12 (16), 2675–2683. 10.4161/cc.25795 23907130 PMC3865057

[B20] Capper D. Jones D. T. W. Sill M. Hovestadt V. Schrimpf D. Sturm D. (2018). DNA methylation-based classification of central nervous system tumours. Nature 555 (7697), 469–474. 10.1038/nature26000 29539639 PMC6093218

[B21] Carpenter E. J. W. P. B. (2012) “Understanding the language of Lys36 methylation at histone H3,” in Nature REVIEWS|MOLECULAR CELL BIOLOGY. 10.1038/nrm3274 PMC3969746 22266761

[B22] Castel D. Philippe C. Calmon R. Le Dret L. Truffaux N. Boddaert N. (2015). Histone H3F3A and HIST1H3B K27M mutations define two subgroups of diffuse intrinsic pontine gliomas with different prognosis and phenotypes. Acta Neuropathol. 130 (6), 815–827. 10.1007/s00401-015-1478-0 26399631 PMC4654747

[B23] Chabot T. Cheraud Y. Fleury F. (2021). Relationships between DNA repair and RTK-mediated signaling pathways. Biochim. Biophys. Acta Rev. Cancer 1875 (1), 188495. 10.1016/j.bbcan.2020.188495 33346130

[B24] Chan K. M. Fang D. Gan H. Hashizume R. Yu C. Schroeder M. (2013). The histone H3.3K27M mutation in pediatric glioma reprograms H3K27 methylation and gene expression. Genes Dev. 27 (9), 985–990. 10.1101/gad.217778.113 23603901 PMC3656328

[B25] Chang H. R. Munkhjargal A. Kim M. J. Park S. Y. Jung E. Ryu J. H. (2018). The functional roles of PML nuclear bodies in genome maintenance. Mutat. Res. 809, 99–107. 10.1016/j.mrfmmm.2017.05.002 28521962

[B26] Chaouch A. Lasko P. (2021). Drosophila melanogaster: a fruitful model for oncohistones. Fly. (Austin). 15 (1), 28–37. 10.1080/19336934.2020.1863124 33423597 PMC7808415

[B27] Chen C. C. L. Deshmukh S. Jessa S. Hadjadj D. Lisi V. Andrade A. F. (2020b). Histone H3.3G34-mutant Interneuron progenitors Co-opt PDGFRA for gliomagenesis. Cell 183 (6), 1617–1633. 10.1016/j.cell.2020.11.012 33259802 PMC7791404

[B28] Chen H. Lorton B. Gupta V. Shechter D. (2017). A TGFβ-PRMT5-MEP50 axis regulates cancer cell invasion through histone H3 and H4 arginine methylation coupled transcriptional activation and repression. Oncogene 36 (3), 373–386. 10.1038/onc.2016.205 27270440 PMC5140780

[B29] Chen K. Y. Bush K. Klein R. H. Cervantes V. Lewis N. Naqvi A. (2020a). Reciprocal H3.3 gene editing identifies K27M and G34R mechanisms in pediatric glioma including NOTCH signaling. Commun. Biol. 3 (1), 363. 10.1038/s42003-020-1076-0 32647372 PMC7347881

[B30] Chew G. L. Bleakley M. Bradley R. K. Malik H. S. Henikoff S. Molaro A. (2021). Short H2A histone variants are expressed in cancer. Nat. Commun. 12 (1), 490. 10.1038/s41467-020-20707-x 33473122 PMC7817690

[B31] Choi J. Kim T. Cho E. J. (2024). HIRA vs. DAXX: the two axes shaping the histone H3.3 landscape. Exp. Mol. Med. 56 (2), 251–263. 10.1038/s12276-023-01145-3 38297159 PMC10907377

[B32] Chou D. M. Adamson B. Dephoure N. E. Tan X. Nottke A. C. Hurov K. E. (2010). A chromatin localization screen reveals poly (ADP ribose)-regulated recruitment of the repressive polycomb and NuRD complexes to sites of DNA damage. Proc. Natl. Acad. Sci. U S A. 107 (43), 18475–18480. 10.1073/pnas.1012946107 20937877 PMC2972950

[B33] Cohen L. R. Z. Meshorer E. (2024). The many faces of H3.3 in regulating chromatin in embryonic stem cells and beyond. Trends Cell Biol. 10.1016/j.tcb.2024.03.003 38614918

[B34] Cohn M. A. D'Andrea A. D. (2008). Chromatin recruitment of DNA repair proteins: lessons from the fanconi anemia and double-strand break repair pathways. Mol. Cell 32 (3), 306–312. 10.1016/j.molcel.2008.10.009 18995829 PMC3753040

[B35] Dabin J. Mori M. Polo S. E. (2023). The DNA damage response in the chromatin context: a coordinated process. Curr. Opin. Cell Biol. 82, 102176. 10.1016/j.ceb.2023.102176 37301060

[B36] Delaney K. Strobino M. Wenda J. M. Pankowski A. Steiner F. A. (2019). H3.3K27M-induced chromatin changes drive ectopic replication through misregulation of the JNK pathway in C. elegans. Nat. Commun. 10 (1), 2529. 10.1038/s41467-019-10404-9 31175278 PMC6555832

[B37] Dottermusch M. Uksul N. Knappe U. J. Erdlenbruch B. Wefers A. K. (2022). An H3F3A K27M-mutation in a sonic hedgehog medulloblastoma. Brain Pathol. 32 (3), e13024. 10.1111/bpa.13024 34747078 PMC9048514

[B38] Downing J. R. Wilson R. K. Zhang J. Mardis E. R. Pui C. H. Ding L. (2012). The pediatric cancer genome Project. Nat. Genet. 44 (6), 619–622. 10.1038/ng.2287 22641210 PMC3619412

[B39] Espinoza Pereira K. N. Shan J Licht J. D. Bennett R. L. (2023). Histone mutations in cancer. Biochem Soc Trans. 51 (5), 1749–63. 10.1042/BST20210567 PMC10657182 37721138

[B40] Fang D. Gan H. Lee J. H. Han J. Wang Z. Riester S. M. (2016). The histone H3.3K36M mutation reprograms the epigenome of chondroblastomas. Science 352 (6291), 1344–1348. 10.1126/science.aae0065 27229140 PMC5460624

[B41] Fang J. Huang Y. Mao G. Yang S. Rennert G. Gu L. (2018). Cancer-driving H3G34V/R/D mutations block H3K36 methylation and H3K36me3-MutSα interaction. Proc. Natl. Acad. Sci. U S A. 115 (38), 9598–9603. 10.1073/pnas.1806355115 30181289 PMC6156674

[B42] Faury D. Nantel A. Dunn S. E. Guiot M. C. Haque T. Hauser P. (2007). Molecular profiling identifies prognostic subgroups of pediatric glioblastoma and shows increased YB-1 expression in tumors. J. Clin. Oncol. 25 (10), 1196–1208. 10.1200/JCO.2006.07.8626 17401009

[B43] Ferrand J. Rondinelli B. Polo S. E. (2020). Histone variants: Guardians of genome integrity. Cells 9 (11), 2424. 10.3390/cells9112424 33167489 PMC7694513

[B44] Filbin M. G. Tirosh I. Hovestadt V. Shaw M. L. Escalante L. E. Mathewson N. D. (2018). Developmental and oncogenic programs in H3K27M gliomas dissected by single-cell RNA-seq. Science 360 (6386), 331–335. 10.1126/science.aao4750 29674595 PMC5949869

[B45] Flavahan W. A. Gaskell E. Bernstein B. E. (2017). Epigenetic plasticity and the hallmarks of cancer. Science 357 (6348), eaal2380. 10.1126/science.aal2380 28729483 PMC5940341

[B46] Fnu S. Williamson E. A. De Haro L. P. Brenneman M. Wray J. Shaheen M. (2011). Methylation of histone H3 lysine 36 enhances DNA repair by nonhomologous end-joining. Proc. Natl. Acad. Sci. U S A. 108 (2), 540–545. 10.1073/pnas.1013571108 21187428 PMC3021059

[B47] Fontebasso A. M. Papillon-Cavanagh S. Schwartzentruber J. Nikbakht H. Gerges N. Fiset P. O. (2014). Recurrent somatic mutations in ACVR1 in pediatric midline high-grade astrocytoma. Nat. Genet. 46 (5), 462–466. 10.1038/ng.2950 24705250 PMC4282994

[B48] Fontebasso A. M. Schwartzentruber J. Khuong-Quang D. A. Liu X. Y. Sturm D. Korshunov A. (2013). Mutations in SETD2 and genes affecting histone H3K36 methylation target hemispheric high-grade gliomas. Acta Neuropathol. 125 (5), 659–669. 10.1007/s00401-013-1095-8 23417712 PMC3631313

[B49] Frey A. Listovsky T. Guilbaud G. Sarkies P. Sale J. E. (2014). Histone H3.3 is required to maintain replication fork progression after UV damage. Curr. Biol. 24 (18), 2195–2201. 10.1016/j.cub.2014.07.077 25201682 PMC4175177

[B50] Funato K. Major T. Lewis P. W. Allis C. D. Tabar V. (2014). Use of human embryonic stem cells to model pediatric gliomas with H3.3K27M histone mutation. Science 346 (6216), 1529–1533. 10.1126/science.1253799 25525250 PMC4995593

[B51] Gessi M. Gielen G. H. Hammes J. Dorner E. Muhlen A. Z. Waha A. (2013). H3.3 G34R mutations in pediatric primitive neuroectodermal tumors of central nervous system (CNS-PNET) and pediatric glioblastomas: possible diagnostic and therapeutic implications? J. Neurooncol 112 (1), 67–72. 10.1007/s11060-012-1040-z 23354654

[B52] Giacomini G. Piquet S. Chevallier O. Dabin J. Bai S. K. Kim B. (2024). Aberrant DNA repair reveals a vulnerability in histone H3.3-mutant brain tumors. Nucleic Acids Res. 52, 2372–2388. 10.1093/nar/gkad1257 38214234 PMC10954481

[B53] Haase S. Banerjee K. Mujeeb A. A. Hartlage C. S. Nunez F. M. Nunez F. J. (2022). H3.3-G34 mutations impair DNA repair and promote cGAS/STING-mediated immune responses in pediatric high-grade glioma models. J. Clin. Invest 132 (22), e154229. 10.1172/JCI154229 36125896 PMC9663161

[B54] Halazonetis T. D. Gorgoulis V. G. Bartek J. (2008). An oncogene-induced DNA damage model for cancer development. Science 319 (5868), 1352–1355. 10.1126/science.1140735 18323444

[B55] Hanahan D. (2022). Hallmarks of cancer: new Dimensions. Cancer Discov. 12 (1), 31–46. 10.1158/2159-8290.CD-21-1059 35022204

[B56] Harutyunyan A. S. Krug B. Chen H. Papillon-Cavanagh S. Zeinieh M. De Jay N. (2019). H3K27M induces defective chromatin spread of PRC2-mediated repressive H3K27me2/me3 and is essential for glioma tumorigenesis. Nat. Commun. 10 (1), 1262. 10.1038/s41467-019-09140-x 30890717 PMC6425035

[B57] Hashizume R. Andor N. Ihara Y. Lerner R. Gan H. Chen X. (2014). Pharmacologic inhibition of histone demethylation as a therapy for pediatric brainstem glioma. Nat. Med. 20 (12), 1394–1396. 10.1038/nm.3716 25401693 PMC4257862

[B58] Huang T. Y. Piunti A. Qi J. Morgan M. Bartom E. Shilatifard A. (2020). Effects of H3.3G34V mutation on genomic H3K36 and H3K27 methylation patterns in isogenic pediatric glioma cells. Acta Neuropathol. Commun. 8 (1), 219. 10.1186/s40478-020-01092-4 33287886 PMC7722426

[B59] Hubner J. M. Muller T. Papageorgiou D. N. Mauermann M. Krijgsveld J. Russell R. B. (2019). EZHIP/CXorf67 mimics K27M mutated oncohistones and functions as an intrinsic inhibitor of PRC2 function in aggressive posterior fossa ependymoma. Neuro Oncol. 21 (7), 878–889. 10.1093/neuonc/noz058 30923826 PMC6620627

[B60] Ito T. Teo Y. V. Evans S. A. Neretti N. Sedivy J. M. (2018). Regulation of cellular Senescence by polycomb chromatin modifiers through distinct DNA damage- and histone methylation-dependent pathways. Cell Rep. 22 (13), 3480–3492. 10.1016/j.celrep.2018.03.002 29590617 PMC5915310

[B61] Jackson S. P. Bartek J. (2009). The DNA-damage response in human biology and disease. Nature 461 (7267), 1071–1078. 10.1038/nature08467 19847258 PMC2906700

[B62] Jain S. U. Khazaei S. Marchione D. M. Lundgren S. M. Wang X. Weinberg D. N. (2020). Histone H3.3 G34 mutations promote aberrant PRC2 activity and drive tumor progression. Proc. Natl. Acad. Sci. U S A 117 (44), 27354–27364. 10.1073/pnas.2006076117 33067396 PMC7959516

[B63] Jang C. W. Shibata Y. Starmer J. Yee D. Magnuson T. (2015). Histone H3.3 maintains genome integrity during mammalian development. Genes Dev. 29 (13), 1377–1392. 10.1101/gad.264150.115 26159997 PMC4511213

[B64] Jenseit A. Camgoz A. Pfister S. M. Kool M. (2022). EZHIP: a new piece of the puzzle towards understanding pediatric posterior fossa ependymoma. Acta Neuropathol. 143 (1), 1–13. 10.1007/s00401-021-02382-4 34762160 PMC8732814

[B65] Jessa S. Mohammadnia A. Harutyunyan A. S. Hulswit M. Varadharajan S. Lakkis H. (2022). K27M in canonical and noncanonical H3 variants occurs in distinct oligodendroglial cell lineages in brain midline gliomas. Nat. Genet. 54 (12), 1865–1880. 10.1038/s41588-022-01205-w 36471070 PMC9742294

[B66] Johnson K. J. Cullen J. Barnholtz-Sloan J. S. Ostrom Q. T. Langer C. E. Turner M. C. (2014). Childhood brain tumor epidemiology: a brain tumor epidemiology consortium review. Cancer Epidemiol. Biomarkers Prev. 23 (12), 2716–2736. 10.1158/1055-9965.EPI-14-0207 25192704 PMC4257885

[B67] Jones C. Baker S. J. (2014). Unique genetic and epigenetic mechanisms driving paediatric diffuse high-grade glioma. Nat. Rev. Cancer 14 (10), 651–661. 10.1038/nrc3811 PMC4747023 25230881

[B68] Jones P. A. Baylin S. B. (2007). The epigenomics of cancer. Cell 128 (4), 683–692. 10.1016/j.cell.2007.01.029 17320506 PMC3894624

[B69] Justin N. Zhang Y. Tarricone C. Martin S. R. Chen S. Underwood E. (2016). Structural basis of oncogenic histone H3K27M inhibition of human polycomb repressive complex 2. Nat. Commun. 7, 11316. 10.1038/ncomms11316 27121947 PMC4853476

[B70] Kallappagoudar S. Yadav R. K. Lowe B. R. Partridge J. F. (2015). Histone H3 mutations--a special role for H3.3 in tumorigenesis? Chromosoma 124 (2), 177–189. 10.1007/s00412-015-0510-4 25773741 PMC4446520

[B71] Kang T. Z. E. Zhu L. Yang D. Ding D. Zhu X. Wan Y. C. E. (2021). The elevated transcription of ADAM19 by the oncohistone H2BE76K contributes to oncogenic properties in breast cancer. J. Biol. Chem. 296, 100374. 10.1016/j.jbc.2021.100374 33548228 PMC7949156

[B72] Kfoury-Beaumont N. Prakasam R. Pondugula S. Lagas J. S. Matkovich S. Gontarz P. (2022). The H3K27M mutation alters stem cell growth, epigenetic regulation, and differentiation potential. BMC Biol. 20 (1), 124. 10.1186/s12915-022-01324-0 35637482 PMC9153095

[B73] Khazaei S. Chen C. C. L. Andrade A. F. Kabir N. Azarafshar P. Morcos S. M. (2023). Single substitution in H3.3G34 alters DNMT3A recruitment to cause progressive neurodegeneration. Cell 186 (6), 1162–1178.e20. 10.1016/j.cell.2023.02.023 36931244 PMC10112048

[B74] Khuong-Quang D. A. Buczkowicz P. Rakopoulos P. Liu X. Y. Fontebasso A. M. Bouffet E. (2012). K27M mutation in histone H3.3 defines clinically and biologically distinct subgroups of pediatric diffuse intrinsic pontine gliomas. Acta Neuropathol. 124 (3), 439–447. 10.1007/s00401-012-0998-0 22661320 PMC3422615

[B75] Klein B. J. Krajewski K. Restrepo S. Lewis P. W. Strahl B. D. Kutateladze T. G. (2018). Recognition of cancer mutations in histone H3K36 by epigenetic writers and readers. Epigenetics 13 (7), 683–692. 10.1080/15592294.2018.1503491 30045670 PMC6224213

[B76] Korshunov A. Capper D. Reuss D. Schrimpf D. Ryzhova M. Hovestadt V. (2016). Histologically distinct neuroepithelial tumors with histone 3 G34 mutation are molecularly similar and comprise a single nosologic entity. Acta Neuropathol. 131 (1), 137–146. 10.1007/s00401-015-1493-1 26482474

[B77] Korshunov A. Remke M. Werft W. Benner A. Ryzhova M. Witt H. (2010). Adult and pediatric medulloblastomas are genetically distinct and require different algorithms for molecular risk stratification. J. Clin. Oncol. 28 (18), 3054–3060. 10.1200/JCO.2009.25.7121 20479417

[B78] Lee C. H. Yu J. R. Granat J. Saldana-Meyer R. Andrade J. LeRoy G. (2019). Automethylation of PRC2 promotes H3K27 methylation and is impaired in H3K27M pediatric glioma. Genes Dev. 33 (19-20), 1428–1440. 10.1101/gad.328773.119 31488577 PMC6771381

[B79] Lee E. Park Y. J. Lindroth A. M. (2024). H3.3-G34W in giant cell tumor of bone functionally aligns with the exon choice repressor hnRNPA1L2. Cancer Gene Ther. 31, 1177–1185. 10.1038/s41417-024-00776-6 38811797 PMC11327103

[B80] Lehnertz B. Zhang Y. W. Boivin I. Mayotte N. Tomellini E. Chagraoui J. (2017). H3(K27M/I) mutations promote context-dependent transformation in acute myeloid leukemia with RUNX1 alterations. Blood 130 (20), 2204–2214. 10.1182/blood-2017-03-774653 28855157

[B81] Leszczynska K. B. Freitas-Huhtamaki A. Jayaprakash C. Dzwigonska M. Vitorino F. N. L. Horth C. (2024). H2A.Z histone variants facilitate HDACi-dependent removal of H3.3K27M mutant protein in pediatric high-grade glioma cells. Cell Rep. 43 (2), 113707. 10.1016/j.celrep.2024.113707 38306270 PMC11026119

[B82] Lewis P. W. Muller M. M. Koletsky M. S. Cordero F. Lin S. Banaszynski L. A. (2013). Inhibition of PRC2 activity by a gain-of-function H3 mutation found in pediatric glioblastoma. Science 340 (6134), 857–861. 10.1126/science.1232245 23539183 PMC3951439

[B83] Lin A. Giuliano C. J. Palladino A. John K. M. Abramowicz C. Yuan M. L. (2019). Off-target toxicity is a common mechanism of action of cancer drugs undergoing clinical trials. Sci Transl Med. 11 (509). 10.1126/scitranslmed.aaw8412 PMC7717492 31511426

[B84] Liu C. Kuang S. Wu L. Cheng Q. Gong X. Wu J. (2023). Radiotherapy and radio-sensitization in H3(K27M) -mutated diffuse midline gliomas. CNS Neurosci. Ther. 29 (7), 1721–1737. 10.1111/cns.14225 37157237 PMC10324372

[B85] Liu I. Jiang L. Samuelsson E. R. Marco Salas S. Beck A. Hack O. A. (2022). The landscape of tumor cell states and spatial organization in H3-K27M mutant diffuse midline glioma across age and location. Nat. Genet. 54 (12), 1881–1894. 10.1038/s41588-022-01236-3 36471067 PMC9729116

[B86] Liu X. Y. Gerges N. Korshunov A. Sabha N. Khuong-Quang D. A. Fontebasso A. M. (2012). Frequent ATRX mutations and loss of expression in adult diffuse astrocytic tumors carrying IDH1/IDH2 and TP53 mutations. Acta Neuropathol. 124 (5), 615–625. 10.1007/s00401-012-1031-3 22886134

[B87] Louis D. N. Perry A. Reifenberger G. von Deimling A. Figarella-Branger D. Cavenee W. K. (2016). The 2016 World Health organization classification of tumors of the central nervous system: a summary. Acta Neuropathol. 131 (6), 803–820. 10.1007/s00401-016-1545-1 27157931

[B88] Louis D. N. Perry A. Wesseling P. Brat D. J. Cree I. A. Figarella-Branger D. (2021). The 2021 WHO classification of tumors of the central nervous system: a summary. Neuro Oncol. 23 (8), 1231–1251. 10.1093/neuonc/noab106 34185076 PMC8328013

[B89] Lowe B. R. Yadav R. K. Henry R. A. Schreiner P. Matsuda A. Fernandez A. G. (2021). Surprising phenotypic diversity of cancer-associated mutations of Gly 34 in the histone H3 tail. Elife 10, e65369. 10.7554/eLife.65369 33522486 PMC7872514

[B90] Lu C. Jain S. U. Hoelper D. Bechet D. Molden R. C. Ran L. (2016). Histone H3K36 mutations promote sarcomagenesis through altered histone methylation landscape. Science 352 (6287), 844–849. 10.1126/science.aac7272 27174990 PMC4928577

[B91] Luijsterburg M. S. de Krijger I. Wiegant W. W. Shah R. G. Smeenk G. de Groot A. J. L. (2016). PARP1 links CHD2-mediated chromatin Expansion and H3.3 deposition to DNA repair by non-homologous end-joining. Mol. Cell 61 (4), 547–562. 10.1016/j.molcel.2016.01.019 26895424 PMC4769320

[B92] Lundegaard C. Lamberth K. Harndahl M. Buus S. Lund O. Nielsen M. (2008). NetMHC-3.0: accurate web accessible predictions of human, mouse and monkey MHC class I affinities for peptides of length 8-11. Nucleic Acids Res. 36 (Web Server issue), W509–W512. 10.1093/nar/gkn202 18463140 PMC2447772

[B93] Mackay A. Burford A. Carvalho D. Izquierdo E. Fazal-Salom J. Taylor K. R. (2017). Integrated molecular Meta-analysis of 1,000 pediatric high-grade and diffuse intrinsic pontine glioma. Cancer Cell 32 (4), 520–537. 10.1016/j.ccell.2017.08.017 28966033 PMC5637314

[B94] Mancarella D. Ellinghaus H. Sigismondo G. Veselinov O. Kuhn A. Goyal A. (2024). Deposition of onco-histone H3.3-G34W leads to DNA repair deficiency and activates cGAS/STING-mediated immune responses. Int. J. Cancer 154 (12), 2106–2120. 10.1002/ijc.34883 38353495

[B95] Maze I. Noh K. M. Soshnev A. A. Allis C. D. (2014). Every amino acid matters: essential contributions of histone variants to mammalian development and disease. Nat. Rev. Genet. 15 (4), 259–271. 10.1038/nrg3673 24614311 PMC4082118

[B96] Mohammad F. Helin K. (2017). Oncohistones: drivers of pediatric cancers. Genes Dev. 31 (23-24), 2313–2324. 10.1101/gad.309013.117 29352018 PMC5795778

[B97] Mohammad F. Weissmann S. Leblanc B. Pandey D. P. Hojfeldt J. W. Comet I. (2017). EZH2 is a potential therapeutic target for H3K27M-mutant pediatric gliomas. Nat. Med. 23 (4), 483–492. 10.1038/nm.4293 28263309

[B98] Molaro A. Young J. M. Malik H. S. (2018). Evolutionary origins and diversification of testis-specific short histone H2A variants in mammals. Genome Res. 28 (4), 460–473. 10.1101/gr.229799.117 29549088 PMC5880237

[B99] Mota M. Sweha S. R. Pun M. Natarajan S. K. Ding Y. Chung C. (2023). Targeting SWI/SNF ATPases in H3.3K27M diffuse intrinsic pontine gliomas. Proc. Natl. Acad. Sci. U S A. 120 (18), e2221175120. 10.1073/pnas.2221175120 37094128 PMC10161095

[B100] Moudgil-Joshi J. Kaliaperumal C. (2021). Letter regarding Louis et al: The 2021 WHO Classification of Tumors of the Central Nervous System: A summary. Neuro Oncol. 23 (12), 2120–2121. 10.1093/neuonc/noab190 34596667 PMC8643450

[B101] Nacev B. A. Feng L. Bagert J. D. Lemiesz A. E. Gao J. Soshnev A. A. (2019). The expanding landscape of 'oncohistone' mutations in human cancers. Nature 567 (7749), 473–478. 10.1038/s41586-019-1038-1 30894748 PMC6512987

[B102] Newhart A. Rafalska-Metcalf I. U. Yang T. Joo L. M. Powers S. L. Kossenkov A. V. (2013). Single cell analysis of RNA-mediated histone H3.3 recruitment to a cytomegalovirus promoter-regulated transcription site. J. Biol. Chem. 288 (27), 19882–19899. 10.1074/jbc.M113.473181 23689370 PMC3707690

[B103] Nikolaev A. Fiveash J. B. Yang E. S. (2020). Combined targeting of mutant p53 and Jumonji family histone demethylase Augments therapeutic Efficacy of radiation in H3K27M DIPG. Int. J. Mol. Sci. 21 (2), 490. 10.3390/ijms21020490 31940975 PMC7014308

[B104] Nunez F. J. Mendez F. M. Kadiyala P. Alghamri M. S. Savelieff M. G. Garcia-Fabiani M. B. (2019). IDH1-R132H acts as a tumor suppressor in glioma via epigenetic up-regulation of the DNA damage response. Sci. Transl. Med. 11 (479), eaaq1427. 10.1126/scitranslmed.aaq1427 30760578 PMC6400220

[B105] Ocasio J. K. Budd K. M. Roach J. T. Andrews J. M. Baker S. J. (2023). Oncohistones and disrupted development in pediatric-type diffuse high-grade glioma. Cancer Metastasis Rev. 42 (2), 367–388. 10.1007/s10555-023-10105-2 37119408 PMC10441521

[B106] O'Hagan H. M. Wang W. Sen S. Destefano Shields C. Lee S. S. Zhang Y. W. (2011). Oxidative damage targets complexes containing DNA methyltransferases, SIRT1, and polycomb members to promoter CpG Islands. Cancer Cell 20 (5), 606–619. 10.1016/j.ccr.2011.09.012 22094255 PMC3220885

[B107] Orii K. E. Lee Y. Kondo N. McKinnon P. J. (2006). Selective utilization of nonhomologous end-joining and homologous recombination DNA repair pathways during nervous system development. Proc. Natl. Acad. Sci. U S A. 103 (26), 10017–10022. 10.1073/pnas.0602436103 16777961 PMC1502498

[B108] Paugh B. S. Qu C. Jones C. Liu Z. Adamowicz-Brice M. Zhang J. (2010). Integrated molecular genetic profiling of pediatric high-grade gliomas reveals key differences with the adult disease. J. Clin. Oncol. 28 (18), 3061–3068. 10.1200/JCO.2009.26.7252 20479398 PMC2903336

[B109] Pfister S. X. Ahrabi S. Zalmas L. P. Sarkar S. Aymard F. Bachrati C. Z. (2014). SETD2-dependent histone H3K36 trimethylation is required for homologous recombination repair and genome stability. Cell Rep. 7 (6), 2006–2018. 10.1016/j.celrep.2014.05.026 24931610 PMC4074340

[B110] Pinto L. Baidarjad H. Entz-Werle N. Van Dyck E. (2021). Impact of chromatin dynamics and DNA repair on genomic stability and treatment resistance in pediatric high-grade gliomas. Cancers (Basel) 13 (22), 5678. 10.3390/cancers13225678 34830833 PMC8616465

[B111] Pitroda S. P. Pashtan I. M. Logan H. L. Budke B. Darga T. E. Weichselbaum R. R. (2014). DNA repair pathway gene expression score correlates with repair proficiency and tumor sensitivity to chemotherapy. Sci. Transl. Med. 6 (229), 229ra42. 10.1126/scitranslmed.3008291 PMC4889008 24670686

[B112] Piunti A. Hashizume R. Morgan M. A. Bartom E. T. Horbinski C. M. Marshall S. A. (2017). Therapeutic targeting of polycomb and BET bromodomain proteins in diffuse intrinsic pontine gliomas. Nat. Med. 23 (4), 493–500. 10.1038/nm.4296 28263307 PMC5667640

[B113] Polotskaia A. Xiao G. Reynoso K. Martin C. Qiu W. G. Hendrickson R. C. (2015). Proteome-wide analysis of mutant p53 targets in breast cancer identifies new levels of gain-of-function that influence PARP, PCNA, and MCM4. Proc. Natl. Acad. Sci. U S A. 112 (11), E1220–E1229. 10.1073/pnas.1416318112 25733866 PMC4371979

[B114] Qin B. Lu G. Chen X. Zheng C. Lin H. Liu Q. (2024). H2B oncohistones cause homologous recombination defect and genomic instability through reducing H2B monoubiquitination in Schizosaccharomyces pombe. J. Biol. Chem. 300, 107345. 10.1016/j.jbc.2024.107345 38718864 PMC11167522

[B115] Rajagopalan K. N. Chen X. Weinberg D. N. Chen H. Majewski J. Allis C. D. (2021). Depletion of H3K36me2 recapitulates epigenomic and phenotypic changes induced by the H3.3K36M oncohistone mutation. Proc. Natl. Acad. Sci. U S A. 118 (9), e2021795118. 10.1073/pnas.2021795118 33619101 PMC7936350

[B116] Rominiyi O. Collis S. J. (2022). DDRugging glioblastoma: understanding and targeting the DNA damage response to improve future therapies. Mol. Oncol. 16 (1), 11–41. 10.1002/1878-0261.13020 34036721 PMC8732357

[B117] Ryall S. Guzman M. Elbabaa S. K. Luu B. Mack S. C. Zapotocky M. (2017). H3 K27M mutations are extremely rare in posterior fossa group A ependymoma. Childs Nerv. Syst. 33 (7), 1047–1051. 10.1007/s00381-017-3481-3 28623522

[B118] Sahu V. Lu C. (2022). Oncohistones: Hijacking the histone code. Annu. Rev. Cancer Biol. 6, 293–312. 10.1146/annurev-cancerbio-070120-102521 36589281 PMC9802661

[B119] Schulte J. D. Buerki R. A. Lapointe S. Molinaro A. M. Zhang Y. Villanueva-Meyer J. E. (2020). Clinical, radiologic, and genetic characteristics of histone H3 K27M-mutant diffuse midline gliomas in adults. Neurooncol Adv. 2 (1), vdaa142. 10.1093/noajnl/vdaa142 33354667 PMC7739048

[B120] Schwartzentruber J. Korshunov A. Liu X. Y. Jones D. T. Pfaff E. Jacob K. (2012). Driver mutations in histone H3.3 and chromatin remodelling genes in paediatric glioblastoma. Nature 482 (7384), 226–231. 10.1038/nature10833 22286061

[B121] Selvam K. Rahman S. A. Li S. (2019). Histone H4 H75E mutation attenuates global genomic and Rad26-independent transcription-coupled nucleotide excision repair. Nucleic Acids Res. 47 (14), 7392–7401. 10.1093/nar/gkz453 31114907 PMC6698655

[B122] Sharda A. Humphrey T. C. (2022). The role of histone H3K36me3 writers, readers and erasers in maintaining genome stability. DNA Repair (Amst) 119, 103407. 10.1016/j.dnarep.2022.103407 36155242

[B123] Shen H. Laird P. W. (2013). Interplay between the cancer genome and epigenome. Cell 153 (1), 38–55. 10.1016/j.cell.2013.03.008 23540689 PMC3648790

[B124] Shi L. Shi J. Shi X. Li W. Wen H. (2018). Histone H3.3 G34 mutations alter histone H3K36 and H3K27 methylation in Cis. J. Mol. Biol. 430 (11), 1562–1565. 10.1016/j.jmb.2018.04.014 29689253 PMC6450091

[B125] Shi L. Wen H. Shi X. (2017). The histone variant H3.3 in transcriptional regulation and human disease. J. Mol. Biol. 429 (13), 1934–1945. 10.1016/j.jmb.2016.11.019 27894815 PMC5446305

[B126] Siddaway R. Canty L. Pajovic S. Milos S. Coyaud E. Sbergio S. G. (2022). Oncohistone interactome profiling uncovers contrasting oncogenic mechanisms and identifies potential therapeutic targets in high grade glioma. Acta Neuropathol. 144 (5), 1027–1048. 10.1007/s00401-022-02489-2 36070144 PMC9547787

[B127] Silveira A. B. Kasper L. H. Fan Y. Jin H. Wu G. Shaw T. I. (2019). H3.3 K27M depletion increases differentiation and extends latency of diffuse intrinsic pontine glioma growth *in vivo* . Acta Neuropathol. 137 (4), 637–655. 10.1007/s00401-019-01975-4 30770999 PMC6546611

[B128] Song H. Hollstein M. Xu Y. (2007). p53 gain-of-function cancer mutants induce genetic instability by inactivating ATM. Nat. Cell Biol. 9 (5), 573–580. 10.1038/ncb1571 17417627

[B129] Sturm D. Witt H. Hovestadt V. Khuong-Quang D. A. Jones D. T. Konermann C. (2012). Hotspot mutations in H3F3A and IDH1 define distinct epigenetic and biological subgroups of glioblastoma. Cancer Cell 22 (4), 425–437. 10.1016/j.ccr.2012.08.024 23079654

[B130] Szenker E. Ray-Gallet D. Almouzni G. (2011). The double face of the histone variant H3.3. Cell Res. 21 (3), 421–434. 10.1038/cr.2011.14 21263457 PMC3193428

[B131] Talbert P. B. Henikoff S. (2010). Histone variants--ancient wrap artists of the epigenome. Nat. Rev. Mol. Cell Biol. 11 (4), 264–275. 10.1038/nrm2861 20197778

[B132] Tatavosian R. Duc H. N. Huynh T. N. Fang D. Schmitt B. Shi X. (2018). Live-cell single-molecule dynamics of PcG proteins imposed by the DIPG H3.3K27M mutation. Nat. Commun. 9 (1), 2080. 10.1038/s41467-018-04455-7 29802243 PMC5970213

[B133] Taylor K. R. Mackay A. Truffaux N. Butterfield Y. Morozova O. Philippe C. (2014). Recurrent activating ACVR1 mutations in diffuse intrinsic pontine glioma. Nat. Genet. 46 (5), 457–461. 10.1038/ng.2925 24705252 PMC4018681

[B134] Timp W. Feinberg A. P. (2013). Cancer as a dysregulated epigenome allowing cellular growth advantage at the expense of the host. Nat. Rev. Cancer 13 (7), 497–510. 10.1038/nrc3486 23760024 PMC4636434

[B135] Udugama M. Hii L. Garvie A. Cervini M. Vinod B. Chan F. L. (2021). Mutations inhibiting KDM4B drive ALT activation in ATRX-mutated glioblastomas. Nat. Commun. 12 (1), 2584. 10.1038/s41467-021-22543-z 33972520 PMC8110556

[B136] Venneti S. Garimella M. T. Sullivan L. M. Martinez D. Huse J. T. Heguy A. (2013). Evaluation of histone 3 lysine 27 trimethylation (H3K27me3) and enhancer of Zest 2 (EZH2) in pediatric glial and glioneuronal tumors shows decreased H3K27me3 in H3F3A K27M mutant glioblastomas. Brain Pathol. 23 (5), 558–564. 10.1111/bpa.12042 23414300 PMC3701028

[B137] Volkova N. V. Meier B. Gonzalez-Huici V. Bertolini S. Gonzalez S. Vohringer H. (2020). Mutational signatures are jointly shaped by DNA damage and repair. Nat. Commun. 11 (1), 2169. 10.1038/s41467-020-15912-7 32358516 PMC7195458

[B138] Voon H. P. J. Hii L. Garvie A. Udugama M. Krug B. Russo C. (2023). Pediatric glioma histone H3.3 K27M/G34R mutations drive abnormalities in PML nuclear bodies. Genome Biol. 24 (1), 284. 10.1186/s13059-023-03122-5 38066546 PMC10704828

[B139] Voon H. P. J. Udugama M. Lin W. Hii L. Law R. H. P. Steer D. L. (2018). Inhibition of a K9/K36 demethylase by an H3.3 point mutation found in paediatric glioblastoma. Nat. Commun. 9 (1), 3142. 10.1038/s41467-018-05607-5 30087349 PMC6081460

[B140] Wan Y. C. E. Leung T. C. S. Ding D. Sun X. Liu J. Zhu L. (2020). Cancer-associated histone mutation H2BG53D disrupts DNA-histone octamer interaction and promotes oncogenic phenotypes. Signal Transduct. Target Ther. 5 (1), 27. 10.1038/s41392-020-0131-0 32296031 PMC7060176

[B141] Wen H. Li Y. Xi Y. Jiang S. Stratton S. Peng D. (2014). ZMYND11 links histone H3.3K36me3 to transcription elongation and tumour suppression. Nature 508 (7495), 263–268. 10.1038/nature13045 24590075 PMC4142212

[B142] Willcockson M. A. Healton S. E. Weiss C. N. Bartholdy B. A. Botbol Y. Mishra L. N. (2021). H1 histones control the epigenetic landscape by local chromatin compaction. Nature 589 (7841), 293–298. 10.1038/s41586-020-3032-z 33299182 PMC8110206

[B143] Wu G. Broniscer A. McEachron T. A. Lu C. Paugh B. S. Becksfort J. (2012). Somatic histone H3 alterations in pediatric diffuse intrinsic pontine gliomas and non-brainstem glioblastomas. Nat. Genet. 44 (3), 251–253. 10.1038/ng.1102 22286216 PMC3288377

[B144] Yadav R. K. Jablonowski C. M. Fernandez A. G. Lowe B. R. Henry R. A. Finkelstein D. (2017). Histone H3G34R mutation causes replication stress, homologous recombination defects and genomic instability in S. pombe. Elife 6, e27406. 10.7554/eLife.27406 28718400 PMC5515577

[B145] Yang S. Zheng X. Lu C. Li G. M. Allis C. D. Li H. (2016). Molecular basis for oncohistone H3 recognition by SETD2 methyltransferase. Genes Dev. 30 (14), 1611–1616. 10.1101/gad.284323.116 27474439 PMC4973290

[B146] Yao Y. Dai W. (2014). Genomic instability and cancer. J. Carcinog. Mutagen 5, 1000165. 10.4172/2157-2518.1000165 25541596 PMC4274643

[B147] You J. S. Jones P. A. (2012). Cancer genetics and epigenetics: two sides of the same coin? Cancer Cell 22 (1), 9–20. 10.1016/j.ccr.2012.06.008 22789535 PMC3396881

[B148] Yusufova N. Kloetgen A. Teater M. Osunsade A. Camarillo J. M. Chin C. R. (2021). Histone H1 loss drives lymphoma by disrupting 3D chromatin architecture. Nature 589 (7841), 299–305. 10.1038/s41586-020-3017-y 33299181 PMC7855728

[B149] Zarghooni M. Bartels U. Lee E. Buczkowicz P. Morrison A. Huang A. (2010). Whole-genome profiling of pediatric diffuse intrinsic pontine gliomas highlights platelet-derived growth factor receptor alpha and poly (ADP-ribose) polymerase as potential therapeutic targets. J. Clin. Oncol. 28 (8), 1337–1344. 10.1200/JCO.2009.25.5463 20142589

[B150] Zhang X. Duan S. Apostolou P. E. Wu X. Watanabe J. Gallitto M. (2024). CHD2 regulates Neuron-glioma interactions in pediatric glioma. Cancer Discov. 14, 1732–1754. 10.1158/2159-8290.CD-23-0012 38767413 PMC11456263

[B151] Zhang X. Fawwal D. V. Spangle J. M. Corbett A. H. Jones C. Y. (2023). Exploring the molecular Underpinnings of cancer-Causing oncohistone mutants using yeast as a model. J. Fungi (Basel) 9 (12), 1187. 10.3390/jof9121187 38132788 PMC10744705

[B152] Zhang Y. Chang J. F. Sun J. Chen L. Yang X. M. Tang H. Y. (2018). Histone H3K27 methylation modulates the dynamics of FANCD2 on chromatin to facilitate NHEJ and genome stability. J. Cell Sci. 131 (12), jcs215525. 10.1242/jcs.215525 29760279

[B153] Zhang Y. Shan C. M. Wang J. Bao K. Tong L. Jia S. (2017). Molecular basis for the role of oncogenic histone mutations in modulating H3K36 methylation. Sci. Rep. 7, 43906. 10.1038/srep43906 28256625 PMC5335568

[B154] Zhao Q. Rank G. Tan Y. T. Li H. Moritz R. L. Simpson R. J. (2009). PRMT5-mediated methylation of histone H4R3 recruits DNMT3A, coupling histone and DNA methylation in gene silencing. Nat. Struct. Mol. Biol. 16 (3), 304–311. 10.1038/nsmb.1568 19234465 PMC5120857

